# TIM-3/Galectin-9 Immune Axis in Colorectal Cancer in Relation to KRAS, NRAS, BRAF, PIK3CA, AKT1 Mutations, MSI Status, and the Cytokine Milieu

**DOI:** 10.3390/ijms26146735

**Published:** 2025-07-14

**Authors:** Błażej Ochman, Anna Kot, Sylwia Mielcarska, Agnieszka Kula, Miriam Dawidowicz, Dorota Hudy, Monika Szrot, Jerzy Piecuch, Dariusz Waniczek, Zenon Czuba, Elżbieta Świętochowska

**Affiliations:** 1 Department of Medical and Molecular Biology, Faculty of Medical Sciences in Zabrze, Medical University of Silesia, 19 Jordana, 41-808 Zabrze, Poland; d201228@365.sum.edu.pl (B.O.); s85876@365.sum.edu.pl (A.K.); d201109@365.sum.edu.pl (S.M.); dorota.hudy@sum.edu.pl (D.H.); 2 Department of Oncological Surgery, Faculty of Medical Sciences in Zabrze, Medical University of Silesia, 41-808 Katowice, Poland; d201070@365.sum.edu.pl (A.K.); d201069@365.sum.edu.pl (M.D.); dwaniczek@sum.edu.pl (D.W.); 3 Department of General and Bariatric Surgery and Emergency Medicine in Zabrze, Faculty of Medical Sciences in Zabrze, Medical University of Silesia, 10 Marii Curie-Skłodowskiej, 41-800 Zabrze, Poland; mszrot@sum.edu.pl (M.S.); jpiecuch@sum.edu.pl (J.P.); 4 Department of Microbiology and Immunology, Faculty of Medical Sciences in Zabrze, Medical University of Silesia, 19 Jordana, 41-808 Zabrze, Poland; zczuba@sum.edu.pl

**Keywords:** colorectal neoplasms, TIM-3, Galectin-9, microsatellite instability (MSI), tumor immune microenvironment, KRAS, NRAS, PIK3CA, BRAF

## Abstract

In this study, we investigated the expression of TIM-3 and Galectin-9 (Gal-9) in colorectal cancer (CRC) and their associations with oncogenic mutations, MSI status, cytokine profiles, and transcriptional data. TIM-3 and Gal-9 protein levels were significantly increased in CRC tissues compared to matched non-tumor margins (*p* < 0.05 and *p* < 0.001, respectively). TIM-3 protein concentration was notably higher in PIK3CA-mutated tumors (*p* < 0.05), while no associations were found with *KRAS*, *NRAS*, *BRAF*, *AKT1*, or MSI status. Multiplex cytokine profiling revealed strong correlations between TIM-3 and Gal-9 levels and key immunomodulatory pathways, including IL-10, IL-17, and chemokine signaling. We also observed significant associations with cytokine subsets involved in protumor activity and immune regulation. Gene set enrichment analysis (GSEA) demonstrated that high TIM-3 and Gal-9 expression was associated with upregulation of cell cycle-related pathways, and downregulation of immune signatures, such as interferon responses and TNF-α/NFκB signaling. These findings suggest that increased TIM-3 and Gal-9 expression reflects a shift toward proliferative activity and immune suppression in the CRC tumor microenvironment, highlighting their potential as biomarkers of immunoevasive tumor phenotypes, especially in PIK3CA-mutant CRC tumors.

## 1. Introduction

Over recent decades, progress in immunology has led to the discovery of novel immune checkpoint molecules, playing a role in shaping current approaches to cancer immunotherapy [1]. Monoclonal antibodies targeting immune checkpoints such as programmed death-1 (PD-1)/programmed death-ligand 1 (PD-L1) and cytotoxic T lymphocyte-associated protein 4 (CTLA-4) have demonstrated remarkable efficacy, including in patients with colorectal cancer (CRC) [2,3]. These therapies enhance the immune system’s capacity to recognize and attack tumor cells and are considered promising for long-term tumor control and lowering the risk of cancer recurrence. Nonetheless, a considerable proportion of patients either fail to respond to these therapies or experience disease progression following an initial response [4]. As a result, extensive research is underway to identify biomarkers and patient subsets that are more likely to benefit from immune checkpoint blockade.

T cell immunoglobulin and mucin domain-containing protein 3 (TIM-3), together with lymphocyte activation gene-3 (LAG-3) and T cell immunoreceptor with Ig and ITIM domains (TIGIT), constitutes the next generation of immune checkpoints [5]. TIM-3, an inhibitory receptor, plays a critical role in maintaining immune tolerance. Initially described as a co-inhibitory molecule involved in regulating Type I immune responses, TIM-3 is expressed on differentiated IFN-γ-producing CD4+ and CD8+ T cells [6]. TIM-3 has also been identified as an important regulator of T cell and apoptosis modulator, particularly affecting CD8+ T cells in the context of chronic infections and cancer [7,8]. Exhausted T cells demonstrate impaired proliferation and diminished effector functions, such as cytokine production and cytotoxic activity, significantly impeding effective antitumor immunity [9]. TIM-3 is expressed on multiple immune cell types, including macrophages, dendritic cells, T lymphocytes, and natural killer (NK) cells [10,11,12]. Its interaction with its primary ligand, Galectin-9 (Gal-9), exerts multifaceted effects on immune regulation and tolerance [13,14]. However, additional ligands also interact with TIM-3, such as phosphatidylserine (PtdSer), carcinoembryonic antigen-related cell adhesion molecule 1 (CEACAM1), and high-mobility group protein B1 (HMGB1), contributing to immune suppression, apoptotic cell clearance, and modulation of T cell immunity [15,16,17]. Therefore, the interaction between TIM-3 and Gal-9 is not exclusive. Both molecules participate in multiple signaling pathways through various receptor-ligand interactions. Nonetheless, Gal-9 has emerged as the most functionally significant ligand of TIM-3, despite the presence of multiple other binding partners. Together, TIM-3 and Gal-9 contribute to the suppression of antitumor immune surveillance [14].

Gal-9, a β-galactoside-binding protein and a member of the galectin family, regulates numerous cellular processes. Due to its strong immunomodulatory activity in various disease settings, Gal-9 has emerged as a promising therapeutic target [18]. Originally identified in rat embryonic kidney tissue as a transmembrane urate transporter and an eosinophil chemoattractant secreted by T cells, Gal-9 is now recognized for its roles in tumor immunology [19,20]. Aberrant expression of Gal-9 in solid malignancies is associated with tumor development and metastasis, with effects on cell proliferation, differentiation, adhesion, communication, and apoptosis [21,22,23,24]. Beyond inducing T cell exhaustion and apoptosis via TIM-3 interaction, Gal-9 can also trigger adaptive immune responses by promoting IL-12 production and fostering dendritic cell maturation and cytokine release [25,26,27]. Moreover, it facilitates the differentiation of regulatory T cells (Tregs) while inhibiting T helper 1 (Th1) and Th17 cells, thereby curbing excessive immune activity and inflammation [11,28,29]. Additionally, Gal-9 modulates other immune receptors, including PD-1, CD40, VISTA, and 4-1BB on T cells, as well as Dectin-1 and CD206 on macrophages, underscoring the complexity of its immunoregulatory functions [26,30,31,32,33,34]. TIM-3 and Gal-9 are increasingly regarded as promising targets for next-generation immunotherapies. However, the molecular determinants, including genetic mutations that govern their expression and functional activity, remain incompletely understood and warrant further investigation.

The NCCN Clinical Practice Guidelines in Oncology recommend testing for seven key biomarkers, including *KRAS*, *NRAS*, *BRAF*, and MSI status, to guide personalized treatment strategies and optimize clinical decision-making in CRC [35]. The mutational profile of CRC tumors, including *KRAS*, *NRAS*, *BRAF*, *PIK3CA*, and *AKT1* gene mutation and MSI status significantly influences the characteristics of the tumor microenvironment (TME) and directly impacts patient prognosis. This also plays a critical role in guiding the selection of appropriate treatment strategies, including targeted therapies and immunotherapeutic approaches. The aim of this study was to quantify the expression levels of the immune checkpoint molecules TIM-3 and Gal-9 in CRC tissues and adjacent non-tumor tissue, and to investigate their associations with key oncogenic mutations, including *KRAS*, *NRAS*, *BRAF*, *PIK3CA*, and *AKT1*, as well as MSI status. Furthermore, we analyzed correlations between these immune checkpoint molecules and the expression of 48 selected cytokines, chemokines, and growth factors within tumor tissues to explore their relationship with the CRC TME.

## 2. Results

### 2.1. TIM3 and Gal-9 Protein Concentrations in CRC Tissue and Surgical Margin Tissue

**Figure 1:**
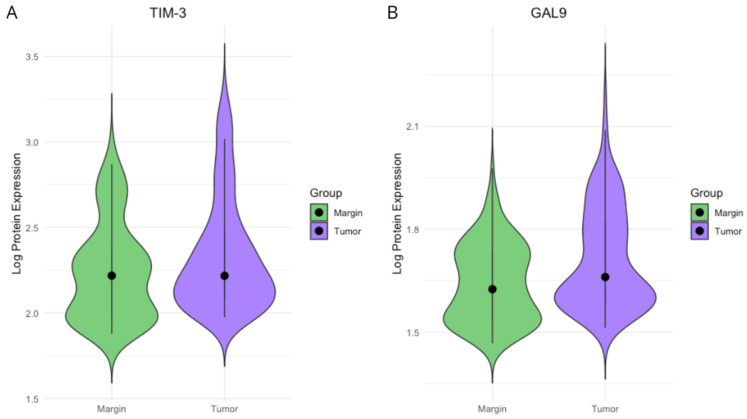
(**A**) TIM-3 protein expression in CRC tissues versus adjacent surgical margins. Wilcoxon signed-rank test (*p* < 0.001). (**B**) Gal-9 protein expression in CRC tissues and matched margins. Wilcoxon signed-rank test indicated a highly significant difference, with a *p*-value < 0.001. All data were subjected to log_10_ transformation prior to analysis.

The concentrations of TIM-3 and Gal-9 proteins were quantitatively assessed in homogenates derived from 131 colorectal cancer (CRC) tissues and 131 adjacent non-tumor tissues. To investigate differences in immune checkpoint protein expression between tumor tissue and adjacent normal tissue, we performed both non-parametric and mixed-effects modeling approaches. Preliminary comparisons using the Wilcoxon signed-rank test revealed significantly higher expression of Gal-9 and TIM-3 in tumor samples compared to matched surgical margins (both *p* < 0.001). To ensure comparability across samples and minimize skewness, protein concentrations were normalized using a base-10 logarithmic transformation. The distribution of TIM-3 and Gal-9 protein expression in both CRC and margin tissues is presented in Figure 1. To account for intra-subject variability, we subsequently fitted linear mixed-effects models with patient ID as a random intercept (See Table S1A,B in Supplementary Material). These models confirmed significantly elevated expression of both proteins in tumor tissue. Gal-9 expression increased by 0.0691 log-units (*p* < 0.0001), while TIM-3 expression increased by 0.0911 log-units (*p* < 0.001). Residual variances were modest, and patient-level random effects captured notable inter-individual variability (Gal-9: SD = 0.085; TIM-3: SD = 0.242). Overall, these findings demonstrate a consistent upregulation of Gal-9 and TIM-3 in the CRC tumor microenvironment.

### 2.2. Associations Between TIM-3 and Gal-9 Expression and KRAS, NRAS, BRAF, PIK3CA, and AKT1 Mutations

Mutational analysis of *KRAS*, *NRAS*, *BRAF*, *PIK3CA*, and *AKT1* genes was performed using real-time PCR. Genetic profiling was conducted on a randomly selected subset of 104 CRC tissue samples from a total cohort of 131 patients diagnosed with CRC. Among the assessed genes, *KRAS* mutations were the most prevalent, identified in 36.53% (*n* = 38) of the analyzed cases. The most frequently occurring *KRAS* mutation variant was located at codons 12/13, representing 76.31% (*n* = 29) of all *KRAS* mutations and accounting for 27.88% of the entire cohort. *NRAS* mutations were detected in 12.50% (*n* = 13) of samples, while alterations in *PIK3CA* and *BRAF* genes were each present in 6.73% (*n* = 7) of cases. Within the PIK3CA-mutated subgroup, the 542/545 hotspot mutation occurred in 85.71% (*n* = 6) of cases. A mutation in the *AKT1* gene was identified in a single case, corresponding to 0.943% of the examined cohort. In the analyzed cohort, 10 tumors (9.615%) exhibited mutations in more than one of the studied genes. Among these, the most common co-mutation involved *KRAS* and *PIK3CA*, identified in 3.85% of tumors (*n* = 4). This was followed by concurrent mutations in *KRAS* and *NRAS*, found in 2.88% of cases (*n* = 3). Single cases were observed with combined mutations in *KRAS* and *BRAF*, *KRAS*, and *AKT1*, and a triple mutation involving *KRAS*, *NRAS*, and *BRAF*, each accounting for 0.96% of the total cohort. Comprehensive frequencies and case numbers for each mutation are provided in Table 1.

**Figure 2:**
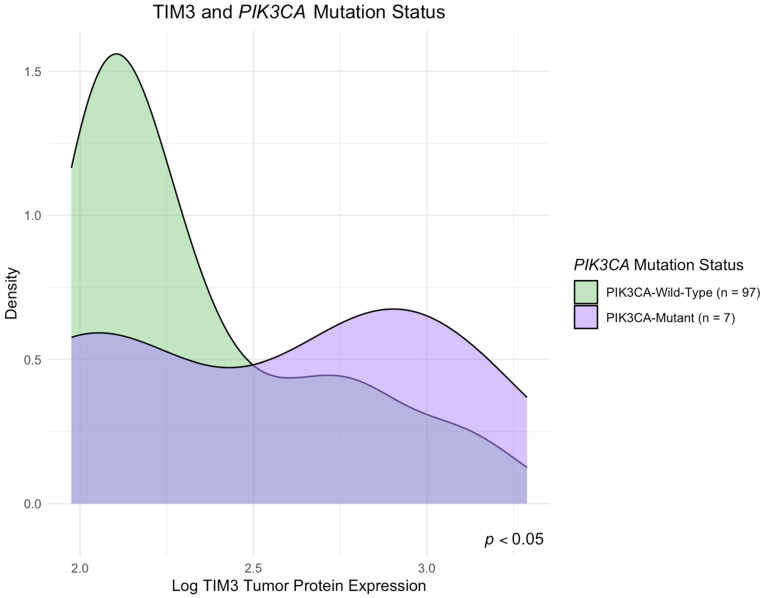
TIM-3 protein concentration according to *PIK3CA* mutation. Density plot shows case counts (n) and significance level determined by the U–Mann–Whitney U test.

A significantly increased protein concentration of the TIM-3 protein was observed in tumors harboring *PIK3CA* mutations (*p* < 0.05) when compared to tumors with wild-type PIK3CA (Figure 2). In contrast, TIM-3 expression did not show statistically significant variation based on the mutational status of *KRAS*, *NRAS*, *BRAF*, or AKT1 genes (*p* > 0.05). Similarly, Gal-9 expression was not significantly associated with any of the evaluated gene mutations (*p* > 0.05). Due to the relatively small number of tumors with multiple gene mutations, we also investigated whether the expression levels of TIM-3 and Gal-9 differed significantly in this whole subgroup compared to tumors lacking mutations in the *KRAS*, *NRAS*, *BRAF*, *PIK3CA*, and *AKT* genes. No statistically significant differences were observed in the concentrations of TIM-3 or Gal-9 between tumors with multiple gene mutations and those without detectable mutations in the analyzed genes (*p* > 0.05). Detailed results of statistical analyses examining TIM-3 and Gal-9 expression in relation to gene mutation status are presented in Supplementary Table S2.

### 2.3. TIM-3 and Gal-9 Proteins Concentration and Selected Cytokine, Chemokine, and Growth Factor Profiles

To gain a more comprehensive understanding of the associations between key immunological molecules within the CRC TME and the expression of TIM-3 and Galectin-9 proteins, we assessed the expression levels of 48 cytokines, chemokines, and growth factors using a multiplex cytokine screening panel. These molecules were classified into functional groups that represent distinct immunological processes, based on gene ontology terms and KEGG pathway annotations. Subsequently, we conducted principal component analysis (PCA) for each cytokine group to reduce dimensionality and capture dominant expression patterns. Additionally, a specific subset of cytokines previously associated with protumor activity was identified through literature review and analyzed separately using PCA, similar to our previous research [36]. Our analysis revealed that the expression levels of TIM-3 and Gal-9 proteins were significantly associated with cytokines, chemokines, and growth factors involved in several key immunological pathways, including IL-10 signaling (respectively *p* < 0.05, R = 0.4809, and *p* < 0.05, R = 0.5036) (Tables S3 and S4, Figure S1), chemokine signaling (TIM3: *p* < 0.01, R = 0.5698, and Gal-9: *p* < 0.01, R = 0.5377) (Tables S5 and S6, Figure S2), IL-17 signaling (TIM3: *p* < 0.05, R = 0.4176, and Gal-9: *p* < 0.05, R = 0.4536) (Tables S7 and S8, Figure S3), NOD-like receptor signaling (TIM3: *p* < 0.05, R = 0.4917, and Gal-9: *p* < 0.05, R = 0.5125) (Tables S9 and S10, Figure S4), macrophage chemotaxis (TIM3: *p* < 0.01, R = 0.5278, and Gal-9: *p* < 0.05, R = 0.4353) (Tables S11 and S12, Figure S5), positive regulation of lymphocyte migration (TIM3: *p* < 0.01, R = 0.6044, and Gal-9: *p* < 0.01, R = 0.5516) (Tables S13 and S14, Figure S6), and protumor activity (TIM3: *p* < 0.05, R = 0.422, and Gal-9: *p* < 0.05, R = 0.4719) (Tables S15 and S16, Figure S7). Within each of these functionally defined cytokine groups, a statistically significant, moderately strong correlation was observed between the expression of TIM-3 and Gal-9 and the first principal component (Factor 1), which accounted for approximately 50–60% of the total variance in each dataset. Detailed statistical results are presented in Table 2. In summary, our findings suggest that TIM-3 and Gal-9 expression is closely linked to distinct cytokine, chemokine, and growth factor expression profiles associated with immunoregulatory, protumor, and chemotactic pathways in the CRC TME. Comprehensive PCA outputs—including eigenvalues, percentages of variance explained for the three principal components, variable loadings after varimax rotation, scree plots, biplots, and correlation matrices—are provided in the Supplementary Materials (Tables S2–S15 and Figures S1–S7). Significant FDR-corrected correlations between Dim.1 and GAL9 expression were found in four pathways: chemokine signaling, IL-10 signaling, NOD-like receptor signaling, and lymphocyte migration (Table S17). For TIM-3, three pathways showed significant associations: chemokine signaling, macrophage chemotaxis, and lymphocyte migration (Table S17).

### 2.4. TIM-3 and Gal-9 Protein Concentration on TNM Staging, Tumor Grade, Primary Tumor Localization, and MSI Status

We investigated the associations between clinical and pathological tumor characteristics and the concentrations of TIM-3 and Gal-9 proteins in the analyzed cohort. The analyzed parameters included TNM classification, clinical stage, histological grade, primary tumor location (right-sided vs. left-sided), tumor-infiltrating lymphocytes (TILs), and MSI status. No significant associations were observed between TIM-3 or Gal-9 protein concentrations and any of the investigated clinicopathological features (*p* > 0.05). Detailed statistical outcomes are provided in Supplementary Tables S18 and S19. Based on our findings, neither TIM-3 nor Gal-9 protein levels demonstrated prognostic relevance. Moreover, TIM-3 and Gal-9 concentrations did not differ significantly between tumors with MSI (*n* = 14) and those with MSS status (*n* = 65), (*p* > 0.05).

### 2.5. Gene Set Enrichment Analysis (GSEA) for High vs. Low Gene Expression of TIM-3 and Gal-9 in CRC Tumors

**Figure 3:**
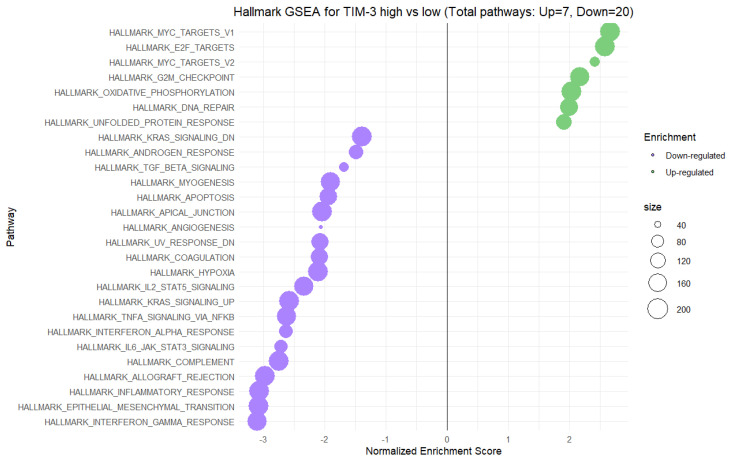
Hallmark gene set enrichment analysis (GSEA) comparing high vs. low expression of TIM-3 in CRC tissues.

To explore the biological pathways and for a deeper understanding of the interactions between the expression of TIM-3 and Gal-9 with the molecular processes occurred in the CRC TME, we have performed GSEA on CRC tumor data. GSEA results were also used to validate our findings from the PCA analysis of cytokine expression data obtained from the cytokine screening panel. In performed GSEA, samples were stratified into TIM-3 and Gal-9 high- and low-expression groups, and enrichment scores were calculated to identify gene sets significantly associated with differential TIM-3 expression. A total of 27 pathways were significantly enriched at a false discovery rate (FDR) < 0.05, including 7 pathways upregulated and 20 pathways downregulated in the high TIM-3 expression group (Figure 3). Among the upregulated pathways, the most significantly enriched gene sets were MYC Targets V1 (normalized enrichment score (NES)~2.6), E2F Targets (NES~2.6), MYC Targets V2 (NES~2.5), and G2M Checkpoint (NES~2.2). These pathways are mostly involved in cell cycle regulation, and cellular proliferation, indicating that increased TIM-3 expression is strongly associated with enhanced proliferative and cell cycle activity. Conversely, pathways significantly downregulated in the TIM-3 high-expression group included Interferon–gamma response (NES~−3.2), Epithelial–Mesenchymal Transition (NES~−3.1), Inflammatory response (NES~−3.2), and Allograft rejection (NES~−3.0) (Figure 3). Additional negatively enriched pathways encompassed TNF-a signaling via NFkB, and Interferon–alpha response, collectively reflecting a broad suppression of immune-related and inflammatory signaling processes in the context of high TIM-3 expression.

**Figure 4:**
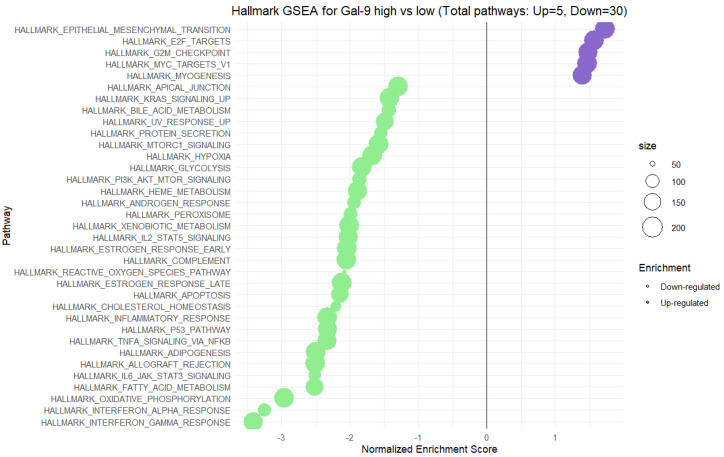
Hallmark gene set enrichment analysis (GSEA) comparing high vs. low expression of TIM-3 in CRC tissues.

As demonstrated in Figure 4, the GSEA identified a total of 35 enriched Hallmark pathways, of which 5 were significantly upregulated and 30 were downregulated in the Gal-9 high-expression group. Among the positively enriched pathways, the highest NES values were observed for Epithelial–Mesenchymal Transition (NES~1.6), E2F targets (NES~1.5), and G2M checkpoint (NES~1.4), suggesting enhanced cellular processes linked to cell cycle progression, proliferation, and epithelia–mesenchymal transition in the Gal-9 high-expressing CRC samples group. Conversely, the majority of pathways exhibited negative enrichment scores, indicating a strong association between high Gal-9 expression and the downregulation of the following biological processes: Interferon–gamma response (NES~−3.3), Interferon–alpha response (NES~−3.2), TNF-a signaling via NFkB (NES~−2.5), and Inflammatory response (NES~−2.4). These results suggest that high Gal-9 expression may correlate with the suppression of type I and II interferon signaling, and immune-related pathways. These data suggest that elevated gene expression of both TIM-3 and Gal-9 expression is associated with enhanced cell cycle activity, and proliferation, alongside a concurrent repression of immune-related pathways.

## 3. Discussion

Over the past decades, numerous studies have demonstrated increased TIM-3 expression across a range of malignancies, including CRC. Based on the findings reported by Zhang et al., the expression levels of TIM-3 and Gal-9 in CRC tissues are significantly associated with disease progression and prognosis following radical resection [37]. Immunohistochemical analyses of 171 patient samples revealed that TIM-3 was highly expressed in 70.18% of tumor tissues, while Gal-9 was highly expressed in only 32.16%. Compared to adjacent non-cancerous tissues, TIM-3 expression was significantly upregulated and Gal-9 was downregulated in tumor samples. The study demonstrated that high TIM-3 expression and low Gal-9 expression were significantly associated with deeper tumor infiltration, vascular invasion, and advanced clinical staging. Survival analyses showed that patients with high TIM-3 or low Gal-9 expression had significantly worse relapse-free survival (RFS) and overall survival (OS). These results suggest that TIM-3 and Gal-9 play opposing roles in colorectal cancer pathogenesis: TIM-3 may promote immune evasion and tumor progression, while Gal-9 may contribute to anti-tumor immunity [37]. The study by Wang et al. further elucidates the functional impact of Gal-9 on anti-tumor immunity in CRC. Their findings demonstrate that Gal-9 expression is significantly reduced in colon tumor tissues compared to adjacent normal mucosa, and this downregulation correlates with poor histological differentiation, lymph node metastasis, and decreased overall survival. Importantly, low Gal-9 expression was associated with markedly reduced infiltration of CD56^+^ natural killer (NK) cells [38]. Based on the study conducted by Yu et al., TIM-3 plays a critical role in the progression of CRC, primarily by enhancing tumor cell proliferation, migration, and invasion. The authors investigated both clinical samples and CRC cell lines, demonstrating that TIM-3 is significantly upregulated in CRC tissues compared to adjacent non-cancerous tissues at both the mRNA and protein levels. Immunohistochemical analysis of CRC cases further revealed that high TIM-3 expression correlated significantly with tumor size, TNM stage, and distant metastasis [38]. In glioblastoma, TIM-3 has emerged as a prominent immune checkpoint molecule, whose overexpression is significantly associated with worse overall survival and has been recognized as an independent predictor of unfavorable prognosis [39,40]. Similarly, in thyroid carcinoma, both TIM-3 and Gal-9 are markedly upregulated compared to normal thyroid tissues [41]. In the context of lung cancer, TIM-3 exhibits a high frequency of expression on both CD4(+) and CD8(+) TILs. Notably, TIM-3 expression on CD4+ T cells has been linked to lymph node metastasis and advanced disease stages [42,43]. Increased TIM-3 expression has also been documented in head and neck squamous cell carcinoma (HNSCC), where it correlates with tumor size, recurrence rates, nodal involvement, and clinical staging, indicating a possible association with adverse prognostic features in HNSCC [44,45]. In gastric cancer, beyond the upregulation of TIM-3, the combined expression profiles of TIM-3 and Gal-9 have been shown to significantly influence patient survival outcomes [46]. Interestingly, in some malignancies, increased TIM-3 or Gal-9 expression has been associated with more favorable clinical outcomes. For example, the presence of TIM-3 on TILs in breast cancer has been reported as an independent predictor of favorable clinical outcomes in breast cancer [47]. Moreover, in advanced triple-negative breast cancer (TNBC), patients exhibiting higher plasma levels of TIM-3 or CTLA-4 have shown better responses to anti-PD-1 immunotherapy combined with VEGFR-2 targeted treatments [48]. Similarly, Gal-9 expression varies substantially between cancer types and their non-cancerous counterparts. Increased expression of Gal-9 has been observed in tumors such as breast cancer, HPV-associated cervical carcinoma, and pancreatic carcinoma [49,50,51], while reduced expression has been reported in gastric, colon, esophageal, melanoma, hepatocellular, renal cell, adrenal, and prostate cancers [38,52,53,54].

In the present study, we identified a significantly increased expression of both TIM-3 and Gal-9 proteins in CRC tissues when compared to non-malignant surgical margin samples (*p* < 0.05 and *p* < 0.001, respectively). The distribution of patients across various clinical stages was relatively balanced, with the majority of cases classified as stage II and III (25.19% and 45.80%, respectively), a pattern that closely mirrors epidemiological data reported in the general population. Notably, no statistically significant associations were identified between TIM-3 or Gal-9 expression levels and clinicopathological variables, including TNM stage, histological tumor grade, or tumor-infiltrating lymphocyte (TIL) density. Increased expression of both proteins was observed across all tumor stages and pathological subgroups, relative to matched normal adjacent tissues. These findings stand in contrast to reports from other studies, which may be partially explained by the clinical characteristics of the patient population enrolled, particularly the stage distribution, as well as methodological differences. Notably, our study employed ELISA-based quantification of protein levels in tumor homogenates, providing insight into the local abundance of these proteins within the TME, rather than relying on transcript-level or immunohistochemical assessments. This methodological distinction could account for the discrepancies, as protein-level measurements better capture the spatial and functional dynamics of the immune landscape within tumors. Research on Gal-9 expression using methodologies similar to ours remains scarce, which underscores the need for further studies to clarify its precise role in the CRC TME and its potential value as a prognostic or therapeutic biomarker.

The RAS family comprises a group of small GTPase proteins ubiquitously expressed in human cells, responsible for transmitting intracellular signals that ultimately regulate cell proliferation. The most clinically relevant members of this family in CRC are *KRAS* and *NRAS*. These proteins are central regulators of signaling cascades that govern essential cellular functions, including proliferation, differentiation, adhesion, programmed cell death, and migration [55,56]. Recent studies have highlighted the multifaceted role of *KRAS* mutations in tumor biology—not only do they intrinsically promote tumorigenesis, but they also modulate the immune microenvironment by driving the production of immunosuppressive cytokines and chemokines through downstream signaling pathways [57]. Moreover, KRAS-mutant tumors frequently harbor concurrent mutations in key tumor suppressor genes, enhancing the tumor’s ability to survive under hypoxic conditions and metabolic stress. These co-mutations contribute to distinct immunological profiles within the TME and can influence tumor response to targeted therapies and immune checkpoint inhibitors (ICIs) [58,59,60]. Additionally, *KRAS* mutations are associated with an increased presence of tumor-associated macrophages (TAMs), further contributing to immunosuppression [61]. *BRAF*, another gene commonly altered in CRC, encodes a serine/threonine kinase involved in the mitogen-activated protein kinase (MAPK) signaling pathway, which regulates cellular proliferation, survival, differentiation, migration, and apoptosis. The *BRAF V600* mutation, in particular, is more prevalent in MSI-high tumors compared to MSI-low tumors and is widely recognized as a biomarker associated with unfavorable clinical outcomes in metastatic CRC (mCRC) [62]. Activating mutations in *PIK3CA*, which encodes the catalytic p110α subunit of class IA phosphoinositide 3-kinases (PI3Ks), contribute to cancer pathogenesis by promoting cell growth, survival, motility, and glucose metabolism. *PIK3CA* mutational status has been investigated as a predictive biomarker for response to MAPK/ERK (MEK) inhibitors and anti-EGFR monoclonal antibodies in CRC. Although early data on its prognostic relevance have been conflicting, recent studies suggest that *PIK3CA* mutations may have a neutral impact on overall survival in CRC patients [63,64]. KRAS mutations are among the most prevalent genetic alterations in colorectal cancer, occurring in approximately 30–50% of cases [65,66]. By contrast, NRAS mutations are relatively uncommon, detected in only 3–10% of CRC cases [67,68]. *KRAS* mutations are most frequently localized to exon 2, particularly at codons 12 and 13, while alterations in exons 3 and 4 are considerably less common [69,70]. For instance, one large study involving 408 CRC tumors reported that *KRAS* exon 2 mutations accounted for 33.1% of cases, whereas *NRAS* mutations were present in 2.4% of tumors [71]. Another study confirmed similar *KRAS* mutation rates, with more than 90% occurring in exon 2 (68.9% at codon 12 and 24.4% at codon 13), and *NRAS* mutations identified in 8.8% of cases [72].

In our cohort of 104 CRC patients, *KRAS* exon 2 mutations were detected at a frequency of 76.31%, aligning with previous observations. Mutations in *KRAS* exons 3 and 4 were both detected at 7.89%, which is higher than previously reported rates of approximately 2.7% and 3.7%. *NRAS* mutations were identified in 12.5% of cases, which also exceeds the frequencies reported in the literature [68,73], with the majority occurring in exon 2 (61.54%) and the remainder in exon 3 (38.46%). These divergences likely reflect population-specific variables, including ethnic background, age distribution, gender, and disease stage. We also screened for *BRAF* exon 15 mutations (including V600E, V600E2, V600D, and V600K) and identified a mutation frequency of approximately 7%, consistent with rates reported in prior studies [74,75]. *PIK3CA* mutations, primarily affecting exons 9 and 20, were found in approximately 7% of cases, a figure slightly lower than the 10–30% range described in the literature [76]. Our analysis revealed that TIM-3 protein concentrations were significantly higher in PIK3CA-mutated CRC tumors compared to *PIK3CA* wild-type counterparts. However, no significant associations were observed between the expression levels of either TIM-3 or Gal-9 and the mutational status of *KRAS*, *NRAS*, *BRAF*, or *AKT* genes. *PIK3CA* encodes the catalytic subunit of PI3K, a key regulator of cellular growth, proliferation, survival, and apoptosis through activation of the AKT-MTOR signaling pathway [77]. Previous research has linked *PIK3CA* mutations to enhanced immune resistance and increased PD-L1 expression in breast and prostate cancers [78]. For example, Ugai et al. reported a higher prevalence of CD274 (PD-L1) positivity in PIK3CA-mutated, PTEN-deficient tumors compared to PIK3CA-wild-type, suggesting that PI3K pathway activation modulates immune checkpoint regulation [79]. Contrary to our findings, He et al. demonstrated a positive association between TIM-3 expression and *BRAF* V600E mutations in CRC, a result further supported by analyses of public datasets, such as TCGA and GEO, which also reported higher TIM-3 expression in BRAF-mutated tumors [80,81]. The associations between genetic mutations and the expression of immune checkpoints such as TIM-3 and Gal-9 remains insufficiently characterized, particularly in CRC. Limited evidence, including a study by Yan et al., has identified a significant positive correlation between *NRAS* expression and TIM-3 levels at both the mRNA and protein levels in lung adenocarcinoma, suggesting a possible link worthy of further investigation [82]. Given the relatively small number of PIK3CA-mutated cases in our cohort—a little less compared to known mutation frequency—our observation of increased TIM-3 expression in this subgroup needs confirmation in larger, independent datasets. The observed upregulation of TIM-3 in PIK3CA-mutated tumors may indicate a functional interaction between the PI3K signaling pathway and TIM-3–driven immunosuppression, which could have significant implications for the development of TIM-3-targeted therapies in CRC.

Microsatellite instability (MSI) represents a pivotal molecular feature in CRC, profoundly influencing tumor biology and guiding therapeutic decision-making. Consequently, CRC can be broadly stratified into two groups based on their mismatch repair (MMR) status: tumors with deficient mismatch repair (dMMR) or MSI-high (MSI-H), which exhibit a high mutational burden (greater than 12 mutations per 10^6^ DNA bases), and those with proficient mismatch repair (pMMR) or microsatellite stable (MSS), characterized by a low mutational burden (less than 8.24 mutations per 10^6^ bases) [83]. Compared to pMMR tumors, MSI-H CRCs display distinct clinicopathologic features, including a proximal colonic location, mucinous histology, poor differentiation, reduced frequency of KRAS and TP53 mutations, and robust infiltration of immune cells, which correlates with a reduced tendency for metastasis. The majority of CRCs (~85%) are pMMR/MSS and generally do not respond to immune checkpoint blockade. In contrast, approximately 15% of CRCs exhibit MSI, marked by a high frequency of small insertions/deletions and point mutations within microsatellite regions. Monoclonal antibodies targeting immune checkpoints such as PD-1, PD-L1, and CTLA-4 have demonstrated significant efficacy in a range of malignancies. In particular, dMMR/MSI-H tumors have emerged as strong predictors of responsiveness to immune checkpoint inhibitors. Their elevated neoantigen load, dense immune infiltration, and high mutational burden collectively contribute to robust immune activation and effective therapeutic responses [84].

Our study did not reveal any significant differences in TIM-3 or Gal-9 protein expression in relation to MSI status. Previous investigations, including those by An et al. and Katagata et al., reported higher TIM-3 expression in MSI CRC compared to MSS tumors [85,86]. However, in our study, TIM-3 protein was quantified via ELISA, while MSI status was assessed immunohistochemically, unlike previous studies that evaluated both variables using immunohistochemistry [85,86]. This methodological difference may partially explain the divergent results. Given the distinct immunological landscape associated with MSI tumors—including upregulated expression of immune checkpoints such as PD-1, PD-L1, and CTLA-4—it could be hypothesized that TIM-3 expression would similarly be increased in MSI CRC. Nevertheless, in our cohort, increased TIM-3 and Gal-9 levels were observed in both MSI and MSS tumors. Understanding the expression dynamics of TIM-3 and Gal-9 in the context of MSI status is of critical importance, particularly given the therapeutic potential of immune checkpoint inhibitors targeting these molecules. Our findings highlight the need for larger-scale, methodologically unified studies to clarify these associations and guide future therapeutic strategies in CRC.

Gal-9 acts through various receptors, exerting numerous functions in immune cells. Endogenous Gal-9 in T-cells likely participates in TCR signal transduction [87]. Gal-9 knockdown in CD4 T-cells reduces their proliferation and cytokine expression upon antibody-mediated activation, with the strongest decrease in IL-17 secretion [88]. On the other hand, exogenous Gal-9 can induce T-cell apoptosis, meaning that the source of Gal-9 expression may be crucial for T lymphocyte function [89]. Importantly, the protein has a strong apoptotic effect on Th1 cells, mainly through TIM-3 interaction [25]. Studies present contradictory results regarding the influence of exogenous Gal-9 on IFN-γ secretion, with some indicating stimulation, and some a decrease in the cytokine levels upon Gal-9 administration [90,91]. Gal-9 has also been shown to promote immune responses in tumors. It was correlated with stronger infiltrations of mature CD208 + DCs, CD8+ T-cells, and CD3+ T-cells in CRC. In addition, the protein expression on NK cells was associated with stronger IFN-γ and TNF-α secretion, with a higher cytotoxic effect than in the case of Gal-9 knockdown in mice [92,93]. Upon interaction between Gal-9 and NK cells, the expression of genes related to immune functions was suppressed, with reduced NK cell IFN-γ production [94]. In melanoma mouse models, Gal-9 increased macrophage numbers, and its tumor-suppressing functions were abrogated in the absence of macrophages [95]. Gal-9 could also promote FGF2 and monocyte chemotactic protein-1 (MCP-1) production in macrophages, but decreased macrophage-derived chemokine (MDC) secretion [26]. TIM-3 is abundantly expressed on dendritic cells, mainly conventional DCs. In CRC, TIM-3 can inhibit T-cell activity through the downregulation of cytokines secreted by DCs [96,97]. In TME, TIM-3 competitively binds to RAGE on DCs, inhibiting pathways related to pattern recognition receptors that normally lead to IL-12 and IFN-γ secretion with subsequent activation of innate immune responses after stimulation with nucleic acids [17,98]. TIM-3 is also expressed on NK cells and can be induced by numerous cytokines, including IL-15, IL-12, and IL-18. In cancer, its expression serves as a marker of NK cell exhaustion [99,100]. Moreover, Gal-9/TIM-3 signaling has been demonstrated to stimulate IL-6, IL-8, and IL-10 secretion in monocytes in the presence of LPS, playing a role in GC progression [101].

To elucidate the immunological context of TIM-3 and its established ligand Gal-9 within the tumor microenvironment, we combined GSEA with PCA performed on our experimental cytokine expression data. Our integrative analysis combining multiplex cytokine profiling, PCA, and GSEA provides novel insights into the immunobiological landscape shaped by TIM-3 and Galectin-9 expression in CRC TME. The PCA identified strong positive correlations between TIM-3 and Gal-9 protein levels and the first principal component (PC1) across multiple cytokine subsets, particularly those involved in chemokine signaling, IL-10 and IL-17 pathways, lymphocyte migration, and macrophage chemotaxis. Notably, PC1 consistently captured variance in the expression of key chemoattractants such as MCP-1, RANTES, IP-10, IL-8, and SDF-1α—molecules known to regulate immune cell trafficking, particularly of monocytes, T cells, and natural killer (NK) cells. These findings suggest that tumors with elevated TIM-3 and Gal-9 expression maintain an active chemokine gradient capable of recruiting various immune cell populations into the TME. However, this apparent immune recruitment is contrasted by our transcriptome-level findings from GSEA. High TIM-3 and Gal-9 expression was associated with strong negative enrichment of multiple immune effector pathways, including interferon-α and interferon-γ responses, TNF-α/NFκB signaling, and broader inflammatory signaling cascades. These transcriptional profiles indicate an overall suppression of innate and adaptive immune activation, consistent with functional exhaustion or exclusion of effector immune responses despite the chemokine-rich environment. This discrepancy between protein-level cytokine data and transcriptional suppression highlights a critical aspect of immune dysregulation in CRC: the physical presence of immune cells may not correlate with their functional capacity. Tumors may thus exhibit immune infiltration in the absence of effective cytotoxic or pro-inflammatory activity—a phenomenon increasingly described as immune dysfunctional state [102].

Taken together, these findings highlighted the dual role of the TIM-3/Gal-9 in modulating the TME. While both molecules are associated with transcriptional repression of pathways involved in effector T cell function and innate immune activation, their expression coincides with the upregulation of chemokines and pro-tumorigenic cytokines that facilitate immune cell trafficking, stromal remodeling, and potentially immune escape. This underscores a complex and dynamic immunological landscape, wherein TIM-3 and Gal-9 may act as central mediators of immune suppression while simultaneously shaping the chemokines’ expression in a manner that favors tumor progression and immune system modulation. These results reinforce the emerging concept of TIM-3 and Gal-9 as interconnected immune checkpoints that not only blunt antitumor immunity but also contribute to the spatial and functional organization of immune infiltrates within the tumor microenvironment, highlighting their potential as therapeutic targets in immune checkpoint blockade strategies.

Despite the novel insights provided by our protein expression profiling of TIM-3 and Gal-9 in CRC, this study has notable limitations. Our analyses are primarily descriptive and correlative, focusing on the quantification of TIM-3 and Gal-9 protein levels and their associations with common oncogenic mutations and selected immunological parameters in CRC tissues. While these findings offer insight into potential molecular patterns, they do not assess the functional consequences of TIM-3 or Gal-9 expression within the CRC TME. Furthermore, no data on patient outcomes or response to therapy were included, and ligand–receptor interactions were not assessed. These limitations should be addressed in future studies aiming to establish the clinical utility of TIM-3 and Gal-9 as biomarkers or therapeutic targets.

## 4. Materials and Methods

### 4.1. Study Group Characteristics

A total of 262 tissue samples were collected and analyzed, including 131 CRC tissue specimens assigned to the study group and 131 tumor-free surgical margin tissues, which served as the control group. The study group consisted solely of tissues with a histopathologically confirmed diagnosis of CRC, while the control group comprised surgical margins without any pathological signs of CRC. Inclusion criteria for the study were as follows: (1) written informed consent from the patient, (2) age over 18 years, and (3) histopathological confirmation of colorectal adenocarcinoma or tumor-free surgical margins. Patients who did not meet these criteria were excluded from the study. The Research Ethics Committee provided approval for the study (PCN/0022/KB1/42/VI/14/16/18/19/20). The median age of patients included in the study was 65 years (±9.22). Most of the tumors (71.76%) were located on the left (distal) side of the colon. Stage III tumors were the most common (45.80%) among the CRC samples, followed by stage II (25.19%). Stage I and stage IV tumors were observed at the same frequency, each accounting for 14.50% of the analyzed specimens. A summary of the clinical and pathological characteristics of the study group is presented in Table 1.

Clinicopathological features of the study group are detailed in Table 3.

### 4.2. Protein Quantification Protocol for TIM-3 and Gal-9

A total of 131 CRC tumor and 131 margin tissues were homogenized according to a previously established protocol [103]. Samples were homogenized in phosphate-buffered solution using a PRO 200 homogenizer (PRO Scientific Inc., Oxford, CT, USA) at 10,000 rpm and further disrupted using ultrasonic sonication (UP100, Hilscher, Hattersheim am Main, Germany). Total protein content was measured using the pyrogallol-red method (Sentinel Diagnostics, Milan, Italy) with a Technicon RA-XTTM analyzer (Technicon Instruments Corporation, Mahopac, NY, USA) at 600 nm and 37 °C. Concentrations of TIM-3 and Gal-9 proteins were quantified using enzyme-linked immunosorbent assay (ELISA) kits (SEH930Hu and SEA309Hu; Cloud Clone, Wuhan, China), following manufacturer protocols. Optimal sample dilutions were established through preliminary assays. The minimal detectable concentrations for the TIM-3 and Gal-9 assays were <31 pg/mL and <33 pg/mL, respectively. Final results were recalculated to the corresponding total protein content and presented as pg/mL of protein.

### 4.3. Assessment of KRAS, NRAS, BRAF, PIK3CA, and AKT Mutation Status

The mutational status of *KRAS*, *NRAS*, *BRAF*, *PIK3CA*, and *AKT* genes was evaluated using real-time polymerase chain reaction (RT-PCR). Detailed procedures were previously described [36]. Genomic DNA was isolated from fresh-frozen CRC tumor samples stored at −80 °C using an automated extraction system and the Mag-Bind Blood & Tissue DNA HDQ 96 Kit (M6399-00), according to the manufacturer’s protocol. DNA concentrations were determined via spectrophotometry, and all samples were diluted to a final concentration of 2 ng/μL, as specified in the RT-PCR kit instruction. Mutational analysis was performed using the CRC-RT48 Mutation Detection Panel for Real-Time PCR (EntroGen, Woodland Hills, CA, USA). Amplification and detection were conducted with the QuantStudio™ 5 Real-Time PCR System (Thermo Fisher Scientific, Waltham, MA, USA). The panel targets mutations in *KRAS* (exons 2, 3, and 4), NRAS (exons 2, 3, and 4), *BRAF* (exon 15), *PIK3CA* (exons 9 and 20), and *AKT1* (exon 4). This approach enabled the identification of specific hotspot mutations, including *KRAS* 12/13, 117, 61, 146, 59; *NRAS* 12/13, 117, 61, 146, 59; *BRAF* V600; *PIK3CA* 542/545, 1047; and *AKT1* E17K (Table 4)

### 4.4. Microsatellite Instability (MSI) Evaluation

MSI status was determined using immunohistochemistry (IHC) on formalin-fixed, paraffin-embedded (FFPE) CRC tissue blocks, following previously established protocols [104,105]. In total, 79 tumor tissue samples were analyzed for MSI status. Tissue sections (4 μm thick) were prepared and stained on a Dako Autostainer Link 48. Slides were deparaffinized, rehydrated, and subjected to antigen retrieval using EnVision Flex Target Retrieval Solution High pH (Dako, Carpinteria, CA, USA) at 95 °C for 20 min. Blocking was followed by incubation with monoclonal primary antibodies targeting mismatch repair proteins: MSH2 (clone G219-1129, Cell Marque, 1:400 for 30 min), MSH6 (clone 44, Cell Marque, 1:100 for 45 min), PMS2 (clone MRQ-28, Cell Marque, 1:50 for 40 min), and MLH1 (clone G168-728, Cell Marque, 1:100 for 40 min). Detection was performed using EnVision FLEX HRP (Dako) and developed with DAB (3,3′-diaminobenzidine), followed by hematoxylin counterstaining and mounting. Nuclear staining in tumor cells was used to assess protein expression, with stromal and inflammatory cells serving as internal controls. Tumors were considered MSI-positive if there was complete loss of nuclear expression in at least one of the following patterns: MLH1 and PMS2, PMS2 alone, MSH2 and MSH6, or MSH6 alone.

### 4.5. Principal Component Analysis (PCA) on Cytokine, Chemokine, and Growth Factor Concentration in CRC Homogenates

The concentrations of cytokines, chemokines, and growth factors in homogenized tissue samples from 77 colorectal cancer (CRC) cases were measured using the Bio-Plex Pro Human Cytokine Screening Panel, 48-Plex (Bio-Rad Laboratories, Hercules, CA, USA), in accordance with the manufacturer’s manual. The obtained concentration data were normalized to total protein level in CRC tissue homogenates and subsequently categorized into functional groups based on Gene Ontology (GO) classifications and KEGG pathway annotations (Table 5) [106,107,108]. Principal Component Analysis (PCA) was applied to the decimal log-transformed, normalized expression data to reduce dimensionality. In the analysis, we used the covariance matrix to extract eigenvalues and eigenvectors, and three principal components (PCs) were chosen for analyses. To enhance interpretability of the factor loadings, component axes were rotated using the varimax rotation. Correlations between these principal components with TIM-3 and Gal-9 protein concentrations were then evaluated. Due to the nature of the data distribution, Spearman’s rank correlation coefficient was used, with statistical significance defined as *p*-values < 0.05. PCA has been performed in R Studio version 4.4.1 using the “factoextra” package.

### 4.6. Gene Set Enrichment Analysis (GSEA) for TIM-3 and Gal-9 Gene Expression on CRC Data

Gene Set Enrichment Analysis (GSEA) has been conducted using the “FieldEffectCrc” dataset, specifically focusing on colorectal cancer (CRC) samples from Cohort A [24] in R Studio version 4.4.1. This dataset comprised Salmon-generated transcript-level quantifications, which were subsequently summarized to the gene level using the tximport package. It included a total of 834 human colorectal tissue samples with three categories: tumor tissue, adjacent normal tissue, and healthy tissue [109]. Data were retrieved using the ExperimentHub package and filtered to include only tumor-derived CRC samples (n = 311). Differential expression analysis was performed using the DESeq function from the DESeq2 package, which accounts for variability in sequencing depth. Normalized gene counts were extracted for downstream analysis. Samples were categorized based on the median expression values of the TIM-3 (ENSG00000135077) and Gal-9 (ENSG00000168961) genes, with a threshold set at 294.795 normalized counts for TIM-3 gene and 7502.06 normalized counts for Gal-9 gene. Samples exceeding this threshold were classified as “high expression” (n = 156), while those below were designated as “low expression” (n = 155). Gene symbols were mapped using the org.Hs.eg.db package, and a ranked gene list was generated from the differential expression results for GSEA input. Enrichment analysis was conducted using hallmark gene sets from the MSigDB (Molecular Signatures Database) collection [110,111], and implemented with the fgsea function from the fgsea package. The analysis included 10,000 permutations and was restricted to gene sets containing between 15 and 400 genes. Only pathways with an adjusted *p*-value < 0.05 were retained and subsequently ranked by their normalized enrichment scores (NES).

### 4.7. Statistical Analyses

The normality of the data was assessed using the Shapiro–Wilk test. The data have been normalized through the use of a decimal logarithmic transformation. To compare protein expression levels (TIM-3 and Gal-9) between tumor tissue and matched surgical margins, we initially employed the Wilcoxon signed-rank test for paired data as a non-parametric approach to assess within-subject differences without assuming normality. To account for inter-individual variability and the paired nature of the measurements, we subsequently used linear mixed-effects models (LMMs) with patient ID as a random intercept. These models allow for robust estimation of fixed effects (tissue type: tumor vs. margin) while adjusting for the correlation structure introduced by repeated measurements within the same subjects. The models were specified as follows: Protein~TissueType + (1 | PatientID), and fitted using restricted maximum likelihood (REML). Degrees of freedom for fixed effects were approximated using Satterthwaite’s method, and *p*-values were reported accordingly. Model residuals were visually inspected to confirm assumptions of homoscedasticity and approximate normality. Correlations between protein concentrations and TNM parameters were evaluated using Kendall’s Tau rank correlation. For examined protein expression and its association with mutation of examined genes we used the Mann–Whitney U test. For examining other correlations, Spearman’s rank correlation was applied. To account for multiple comparisons in the correlation analyses between principal component scores, TIM-3 and Gal-9 protein expression, we applied the Benjamini–Hochberg procedure to control the false discovery rate (FDR). Specifically, for each pathway-level PCA, we computed Spearman correlation coefficients between Gal-9 and TIM-3, and the first principal components (PC1), reflecting dominant axes of protein expression variation within the pathway. Adjusted q-values were reported alongside Spearman ρ and nominal *p*-values. Associations were considered statistically significant only if q < 0.05. This correction was applied independently for each PCA/pathway analysis. Results were summarized in Supplementary Table S17 with significant correlations explicitly flagged. Statistical significance was set at *p* < 0.05. The statistical analyses were performed in R Studio 4.4.1.

## 5. Conclusions

TIM-3 expression correlated with *PIK3CA* mutation status, while both proteins showed robust associations with cytokine networks linked to immunoregulation, chemotaxis, and protumor activity. Gene set enrichment analyses further supported a dual role for TIM-3 and Gal-9 in promoting tumor growth while attenuating antitumor immune responses. These results highlight the TIM-3/Gal-9 axis as a potential biomarker of immune escape and proliferative activity in CRC, warranting further investigation as a therapeutic target in immunomodulatory strategies.

## Figures and Tables

**Table 1:** Frequencies of KRAS, NRAS, BRAF, PIK3CA, and AKT1 gene mutations in the analyzed CRC cohort.

Gene	Mutation Status		Percent (%)	
*n* = 104	Wild-Type	Mutant	Percent Among Mutation of the One Gene	Percent Among All Group
*KRAS*	66	38		36.53%
*KRAS-117-STATUS*	100	4	10.52%	3.84%
*KRAS-12/13-STATUS*	75	29	76.31%	27.88%
*KRAS-59-STATUS*	101	3	7.89%	2.88%
*KRAS-146-STATUS*	102	2	5.26%	1.92%
*KRAS-61-STATUS*	101	3	7.89%	2.88%
*NRAS*	91	13		12.50%
*NRAS-12-13-STATUS*	96	8	61.54%	7.69%
*NRAS-61-STATUS*	99	5	38.46%	4.80%
*PIK3CA*	97	7		6.73%
*PIK3CA 542/545*	99	6	85.71%	5.77%
*PIK3CA 1047*	103	1	14.28%	0.961%
*BRAF*	97	7		6.73%
*AKT*	103	1		0.961%
Combined gene mutations		9		9.615%
*KRAS + NRAS*		3		2.88%
*KRAS + PIK3CA*		4		3.846%
*KRAS + BRAF*		1		0.961%
*KRAS + AKT1*		1		0.961%
*KRAS + NRAS + BRAF*		1		0.961%

**Table 2:** Principal component analysis (PCA) results for cytokines, chemokines, and growth factors measured using a multiplex cytokine screening panel. Cytokine subsets were defined based on Gene Ontology (GO) terms, KEGG pathway annotations, and—specifically for the protumor cytokine group—literature review. For each subset, the table lists the molecules that showed the strongest contribution (i.e., highest loading values) to Principal Component 1 (PC1), which explained the largest proportion of variance within the given group. Spearman’s correlation analysis was performed to evaluate the association between PC1 scores and the expression levels of TIM-3 and Gal-9. The table presents the correlation coefficients (R-values) and corresponding *p*-values.

Cytokine Subset	Top-Contributing Variables to Principal Component 1 (Based on Factor Loadings)	TIM3		Gal-9	
		*p*-Value	R-Value	*p*-Value	R-Value
Chemokine signaling	RANTES, SDF-1a, MCP1, CTACK, IL-8 IP-10	***	0.5698	***	0.5377
Interleukin-10 signaling	IL-18, IL-8, RANTES, TNF-a, MCP1, MIP-1b, G-CSF, IP-10, MIP-1a, IL-10	**	0.4809	**	0.5036
Interleukin-17 signaling pathway	TNF-a, IL-4, MCP3, Eotaxin, IL-8, GRO-a, MCP1, GM-CSF, IL-17,	**	0.4176	**	0.4536
NOD-Like receptor signaling pathway	IL-18, IL-8, IFN-a2, RANTES, TNF-a	**	0.4917	**	0.5125
Macrophage chemotaxis	RANTES, MCP1, IL-8, MIG, IP-10	***	0.5278	**	0.4353
Positive regulation of lymphocyte migration	SDF-1a, RANTES, CTACK, MIP-1a, IP-10	***	0.6044	***	0.5516
Protumor cytokines	IL-18, TNF-a, IL-8, RANTES, MCP3, MCP1	**	0.4220	**	0.4719

Statistically significant correlations were marked as *p* < 0.05 (**) or *p* < 0.01 (***).

**Table 3:** Characteristics of the patient group.

	Female	Male	All
Age	66.5 ± 9.64	65.0 ± 8.75	65 ± 9.22
Tumor localization			
Left-side tumor	43	51	94 (71.76%)
Right-side tumor	18	18	36 (27.48%)
T parameter			
T1	2	5	7 (5.34%)
T2	12	9	21 (16.03%)
T3	41	46	87 (66.41%)
T4	8	8	16 (12.21%)
N parameter			
N0	25	30	55 (41.98%)
N1	26	28	54 (41.22%)
N2	11	11	22 (16.79%)
M parameter			
M0	55	56	111 (84.73%)
M1	7	13	20 (15.27%)
TNM Stage			
I	10	9	19 (14.50%)
II	15	18	33 (25.19%)
III	30	30	60 (45.80%)
IV	7	12	19 (14.50%)
Histological Grading			
High	10	12	22 (16.79%)
Low	52	38	109 (83.21%)
MSI Status (*n* = 79)			
MSS tumors	30	35	65 (82.28%)
MSI tumors	9	5	14 (17.72%)

**Table 4:** The relevant hotspot mutations detected by the RT-PCR assay across the KRAS, NRAS, BRAF, PIK3CA, and AKT1 genes, with the exon location, specific amino acid substitutions, corresponding nucleotide changes, and their COSMIC database identifiers.

Gene	Exon	Amino Acid Change	Nucleotide Change	Cosmic ID
KRAS	2	G12A	c.35G>C	522
		G12D	c.35G>A	521
		G12R	c.34G>C	518
		G12C	c.34G>T	516
		G12S	c.34G>A	517
		G12V	c.35G>T	520
		G13D	c.38G>A	532
	3	A59T	c.175G>A	546
		A59E	c.176C>A	547
		A59G	c.176C>G	28518
		Q61H	c.183A>C	554
		Q61H	c.183A>T	555
		Q61L	c.182A>T	553
		Q61R	c.182A>G	552
	4	K117N	c.351A>C	19940
		K117N	c.351A>T	28519
		K117R	c.350A>G	4696722
		K117E	c.349A>G	-
		A146T	c.436G>A	19404
		A146P	c.436G>C	19905
		A146V	c.437C>T	19900
NRAS	2	G12D	c.35G>A	564
		G12S	c.34G>A	563
		G12C	c.34G>T	562
		G13R	c.37G>C	569
		G13V	c.38G>T	574
	3	A59T	c.175G>A	578
		A59D	c.176C>A	253327
		Q61K	c.181C>A	580
		Q61L	c.182A>T	583
		Q61R	c.182A>G	584
		Q61H	c.183A>C	586
		Q61H	c.183A>T	585
	4	K117R	c.350A>G	-
		A146T	c.436G>A	27174
BRAF	15	V600E	c.1799T>A	476
		V600E2	c.1799-1800TG>AA	-
		V600D	c.1799-1800TG>AT	477
		V600K	c.1798-1799GT>AA	473
PIK3CA	9	E542K	c.1624G>A	760
		E545K	c.1633G>A	763
		E545Q	c.1633G>C	27133
	20	H1047R	c.3140A>G	775
		H1047L	c.3140A>T	776
AKT1	4	E17K	c.49G>A	33765

**Table 5:** Cytokines, chemokines, and growth factor sets assigned to the appropriate Gene Ontology (GO) terms and Kyoto Encyclopedia of Genes and Genomes (KEGG) annotations.

Process Name	Cytokines Involved	Origin
Positive regulation of immune system process	MIF, SCF, MCP1, SDF-1a, VEGFA, MCP3, MCSF, MIP-1a, IL-1a, IL-18, IL-6, RANTES, IL-5, TNF-b, LIF, IL-2, IL-1b, IL-7, IFN-g, IL-13, TNF-a, IL-10, IL-8, IL-4, IP-10, IL-15, IL-2Ra, IL-16, CTACK, IL-12p40, MIP-1b, IL-17	GO
Chemokine signaling pathway	IL-8, MCP1, SDF-1a, GRO-a, IP-10, RANTES, MIP-1a, CTACK, Eotaxin, MCP3, MIP-1b	KEGG
Positive regulation of lymphocyte migration	IP-10, SDF-1a, MIP-1a, CTACK, MIP-1b, MCP3, RANTES	GO
Macrophage chemotaxis	MCP1, IL-8, Eotaxin, MIG, IP-10, GRO-a, MIP-1b, MCP3, RANTES, IL-1b	GO
PI3K-Akt signaling pathway	IL-2Ra, bNGF, IL-2, IL-3, IL-4, SCF, MCSF, IFN-a2, HGF, G-CSF, IL-7, PDGF-bb, BasicFGF, IL-6, VEGFA	KEGG
Leukocyte activation	IL-4, IL-15, IFN-g, SCF, IL-2Ra, IL-8, MCSF, IL-13, IL-18, MIP-1a, RANTES, IL-10, GM-CSF, IL-9, IL-7, IFN-a2, IL-2, IL-6, TNF-a	GO
Inflammatory response	IL-9, CTACK, Eotaxin, MCP1, IFN-a2, IL-1Ra, IL-2Ra, IFN-g, IL-15, IL-1a, IL-6, IL-17, IL-4, MCP3, MIP-1a, IL-18, CTACK, MIF, TNF-a, RANTES, MCSF, MIG, IL-1b, IL-5, IL-10, IL-8, IL-13, IP-10, MIP-1b	GO
Interleukin-10 signaling	MCP1, MCSF, IL-8, IL-18, IL-6, GM-CSF, LIF, IL-10, IL-1Ra, IL-1a, IP-10, GRO-a, MIP-1a, IL-1b, MIP-1b, G-CSF, RANTES, TNF-a	KEGG
NOD-like receptor signaling pathway	IL-8, TNF-a, MCP1, IFN-a2, GRO-a, IL-6, IL-1b, RANTES, IL-18	KEGG
Protumor cytokines	IL-1a, IL-1b, IL-6, IL-8, IL-17, IL-18, TNF-a, RANTES, MCP1, MCP3, MIF, G-CSF, GM-CSF	Own literature review
IL-17 signaling	MCP1, IL-8, IL-1b, IL-4, IL-5, IL-6, IL-13, IL-17, GM-CSF, IFN-g, G-CSF, TNF-a, MCP3, Eotaxin, GRO-a, IP-10	KEGG
Positive regulation of cytokine production	IL-9, IL-12p70, GM-CSF, IL-10, HGF, IL-2, IL-15, IL-1b, IL-18, IFN-g, IL-7, IL-4, TNF-a, IL-17, MIF, TNF-b, IL-16, IL-13, MIP-1a, IL-1a, IL-6	GO

## Data Availability

GSEA data: Dampier CH (2020). “FieldEffectCrc: Tumor, tumor-adjacent normal, and healthy colorectal transcriptomes as SummarizedExperiment objects”. https://bioconductor.org/packages/release/data/experiment/html/FieldEffectCrc.html (accessed on 10 April 2025). Experimental data can be shared upon request.

## Supplementary Materials

The following supporting information can be downloaded at: https://www.mdpi.com/article/10.3390/ijms26146735/s1.

## Abbreviations

BasicFGF	Basic Fibroblast Growth Factor
CCL	Chemokine (C-C motif) ligand
CRC	Colorectal cancer
CSF	Colony-stimulating factor
CTACK	Cutaneous T Cell-Attracting Chemokine
CTLA-4	Cytotoxic T lymphocyte antigen 4
CXCL	Chemokine (C-X-C motif) ligand
EGFR	Epidermal growth factor receptor
Eotaxin	Eotaxin (CCL11)
FDR	False discovery rate
G-CSF	Granulocyte Colony-Stimulating Factor
Gal-9	Galectin-9
GM-CSF	Granulocyte-Macrophage Colony-Stimulating Factor
GO	Gene Ontology
GRO-α	Growth-Regulated Oncogene Alpha
GSEA	Gene Set Enrichment Analysis
HGF	Hepatocyte growth factor
ICB	Immune checkpoint blockade
IFN-γ	Interferon gamma
IL	Interleukin
IP-10	Interferon Gamma-Induced Protein 10
KEGG	Kyoto Encyclopedia of Genes and Genomes
KRAS	Kirsten rat sarcoma viral oncogene homolog
LAG-3	Lymphocyte activation gene-3
MAPK	Mitogen-activated protein kinase
MCP-1/MCP-3	Monocyte Chemoattractant Protein 1/3
MCSF	Macrophage Colony-Stimulating Factor
M-CSF	Macrophage colony-stimulating factor
MIF	Macrophage Migration Inhibitory Factor
MIG	Monokine Induced by Gamma Interferon
MIP-1α/MIP-1β	Macrophage Inflammatory Protein 1 Alpha/Beta
MMR	Mismatch repair
MSI	Microsatellite instability
MSS	Microsatellite stable
NRAS	Neuroblastoma RAS viral oncogene homolog
PCA	Principal Component Analysis
PD-1	Programmed cell death protein 1
PD-L1	Programmed death-ligand 1
PDGF-BB	Platelet-Derived Growth Factor BB
PI3K	Phosphoinositide 3-kinase
RANTES	Regulated on Activation, Normal T Cell Expressed and Secreted (CCL5)
SCF	Stem Cell Factor
SDF-1α	Stromal Cell-Derived Factor 1 Alpha
TAMs	Tumor-associated macrophages
TCGA	The Cancer Genome Atlas
TGF-β	Transforming growth factor beta
TILs	Tumor-infiltrating lymphocytes
TIM-3	T cell immunoglobulin and mucin-domain containing-3
TME	Tumor microenvironment
TNF-α	Tumor necrosis factor alpha
TNF-α/TNF-β	Tumor Necrosis Factor Alpha/Beta
TRAIL	TNF-related apoptosis-inducing ligand
VCAM-1	Vascular cell adhesion protein 1
VEGFA	Vascular Endothelial Growth Factor A
