# Supplementary material for: TIM-3/Galectin-9 Immune Axis in Colorectal Cancer in Relation to KRAS, NRAS, BRAF, PIK3CA, AKT1 Mutations, MSI Status, and the Cytokine Milieu
Source: Int J Mol Sci. 2025 Jul 14;26(14):6735. doi: 10.3390/ijms26146735 (PMC12295348; doi:10.3390/ijms26146735)
Supplement: Supplementary file 1 [file ijms-26-06735-s001.zip › ijms-3627231-supplementary.pdf]

Article

TIM-3/Galectin-9 Immune Axis in Colorectal Cancer in Relation to KRAS, NRAS, BRAF, PIK3CA, AKT1 Mutations, MSI Status, and the Cytokine Milieu

Supplementary Materials

Błażej Ochman [1], Anna Kot [1], Sylwia Mielcarska [1], Agnieszka Kula [2], Miriam Dawidowicz [2], Dorota Hudy [1], Monika Szrot [3], Jerzy Piecuch [3], Dariusz Waniczek[2], Zenon Czuba [4], and Elżbieta Świętochowska [1,*]

[1] Department of Medical and Molecular Biology, Faculty of Medical Sciences in Zabrze, Medical University of Silesia, 19 Jordana, 41-808 Zabrze, Poland; d201228@365.sum.edu.pl (B.O.); s85876@365.sum.edu.pl (A.K.); d201109@365.sum.edu.pl (S.M.); dorota.hudy@sum.edu.pl (D.H.)

[2] Department of Oncological Surgery, Faculty of Medical Sciences in Zabrze, Medical University of Silesia, 41-808 Katowice, Poland; d201070@365.sum.edu.pl (A.K.); d201069@365.sum.edu.pl (M.D.); dwaniczek@sum.edu.pl (D.W.)

[3] Department of General and Bariatric Surgery and Emergency Medicine in Zabrze, Faculty of Medical Sciences in Zabrze, Medical University of Silesia, 10 Marii Curie-Skłodowskiej, 41-800 Zabrze, Poland; mszrot@sum.edu.pl (M.S.); jpiecuch@sum.edu.pl (J.P.)

[4] Department of Microbiology and Immunology, Faculty of Medical Sciences in Zabrze, Medical University of Silesia, 19 Jordana, 41-808 Zabrze, Poland; zczuba@sum.edu.pl (Z.C.)

* Correspondence: eswietochowska@sum.edu.pl

Protein	Fixed Effect	Estimate (β)	Standard Error	Degrees of Freedom	p-Value
GAL-9	Intercept (Margin)	1.6417	0.0114	219.72	< 0.0001 (***)
GAL-9	Tumor vs. Margin	0.0691	0.0122	130.00	8.9×10^{-8} (***)
TIM-3	Intercept (Margin)	2.2532	0.0273	191.33	< 0.0001 (***)
TIM-3	Tumor vs. Margin	0.0911	0.0244	130.00	0.000283 (***)

Table S1A. Linear mixed-effects model results comparing Gal-9 and TIM-3 expression in tumor tissue and matched surgical margins - Fixed Effects. Random Effects

Protein	Random Effect	Variance	Standard Deviation
GAL-9	Patient ID	0.0073	0.0853
GAL-9	Residual	0.0097	0.0986
TIM-3	Patient ID	0.0584	0.2416
TIM-3	Residual	0.0391	0.1976

Table S1B. Linear mixed-effects model results comparing Gal-9 and TIM-3 expression in tumor tissue and matched surgical margins - Random Effects.

Gene	TIM-3	GAL9
n = 106	p-value	p-value
**KRAS**	0.6993	0.4158
KRAS-117-STATUS	0.2937	0.8981
KRAS-12/13-STATUS	0.8088	0.533
KRAS-59-STATUS	0.1608	0.1359
KRAS-146-STATUS	0.1448	0.7378
KRAS-61-STATUS	0.2763	0.9687
**NRAS**	0.4125	0.6159
NRAS-12-13-STATUS	0.8582	0.707
NRAS-61-STATUS	0.3002	0.7647
**PIK3CA**	0.04931	0.316
**BRAF**	0.5379	0.475
**AKT**	0.3727	0.556
**Multimutated tumor group vs non-mutant tumor group**	0.3023	0.351

Table S2. Analysis between TIM-3, Gal-9 proteins concentration and KRAS, NRAS, PIK3CA, and BRAF mutations. P-value from U-Mann-Whitney test.

	eigenvalue	Variance (%)	Cumulative variance (%)
PCA Factor 1	8.611004637	47.83891465	47.83891
PCA Factor 2	2.687123910	14.92846617	62.76738
PCA Factor 3	1.829579392	10.16432996	72.93171

Table S3. Eigenvalue and the percentage of explained variance for 3 factors (principal components) from the PCA for Positive regulation of Interleukin-10 signaling set of cytokines.

Variable	Factor 1	Factor 2	Factor 3
Interleukin-10 signaling *set of cytokines*			
MCP1	0.7869800	0.37355484	-0.002219059
MCSF	0.6637487	-0.50483230	0.142245924
IL_8	0.8560832	-0.09170172	0.065157464
IL_18	0.8661246	0.14622942	-0.139458368
IL_6	0.5566036	0.14813417	-0.192848487
GM_CSF	0.5349675	-0.09688447	-0.558820512
LIF	0.6571635	-0.42200836	-0.343020899
IL_10	0.6923934	-0.25881093	-0.082599244
IL_1Ra	0.4481225	-0.27209494	0.754780373
IL_1a	0.5528370	-0.65298284	0.210871686
IP_10	0.7131655	0.27335573	0.024681281
GRO_a	0.5507634	-0.14158316	-0.639896239
MIP_1a	0.7057658	0.64457001	0.126732160
IL_1b	0.5118046	-0.62959657	0.250438415
MIP_1b	0.7494217	0.19145569	0.394903648
G_CSF	0.7281326	0.56925010	0.141078421
RANTES	0.8479042	0.37770063	0.072129385
TNF-a	0.8195093	-0.27589101	-0.166186089

Table S4. Loadings of 3 factors (PCA Factors) after varimax rotation. Coordinates for the variables for Positive regulation of Interleukin-10 signaling set of cytokines.

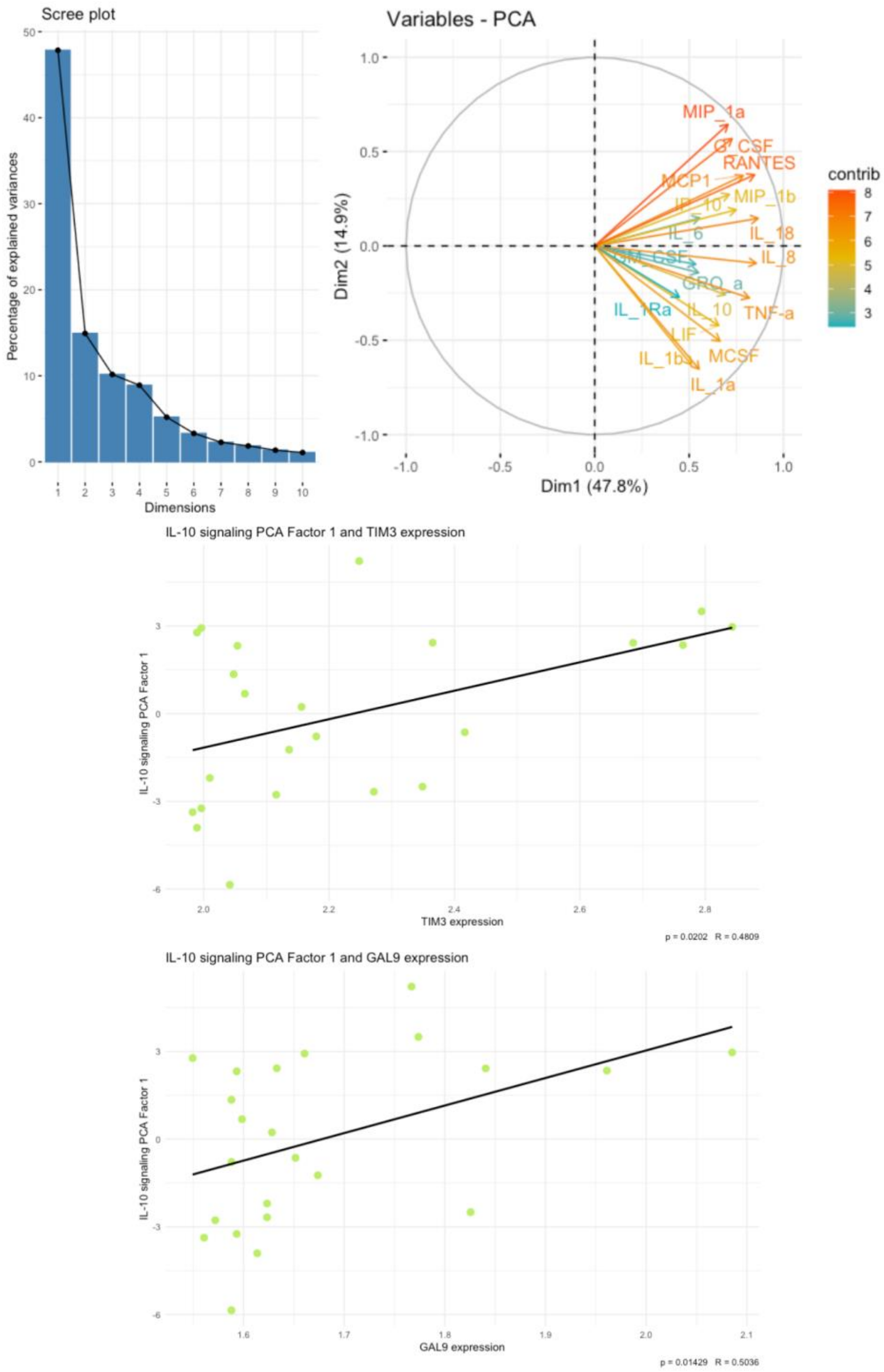

Figure S1. Scree plot, biplot, and correlation plot for Interleukin-10 signaling *set of cytokines* and TIM-3, and Gal-9 expression. The green dots on the plot represent data points from PCA used in the analysis of the correlation between PCA Factor 1 and the TIM-3 and Gal-9 protein expressions.

	eigenvalue	Variance (%)	Cumulative variance (%)
PCA Factor 1	6.82683801	62.0621637	62.06216

PCA Factor 2	1.64744024	14.9767295	77.03889
PCA Factor 3	0.73678001	6.6980001	83.73689

Table S5. Eigenvalue and the percentage of explained variance for 3 factors (principal components) from the PCA for chemokine signaling pathway.

Variable	Factor 1	Factor 2	Factor 3
Positive regulation of lymphocyte chemotaxis			
IL_8	0.7972650	-0.1195239	0.18286807
MCP1	0.8591613	-0.2539846	-0.30293279
SDF-1a	0.8831675	0.0992313	0.19141173
GRO_a	0.5947239	0.5197848	-0.47352241
IP_10	0.7957750	-0.1987635	-0.33487987
RANTES	0.8956955	-0.3086240	-0.11809800
MIP_1a	0.7598444	-0.4580555	-0.05905718
CTACK	0.8323854	0.4389071	0.15162745
Eotaxin	0.7784978	0.2712402	0.29100140
MCP3	0.7321381	0.6462655	0.09493958
MIP_1b	0.6866765	-0.5100810	0.32317712

Table S6. Loadings of 3 factors (PCA Factors) after varimax rotation. Coordinates for the variables for chemokine signaling pathway.

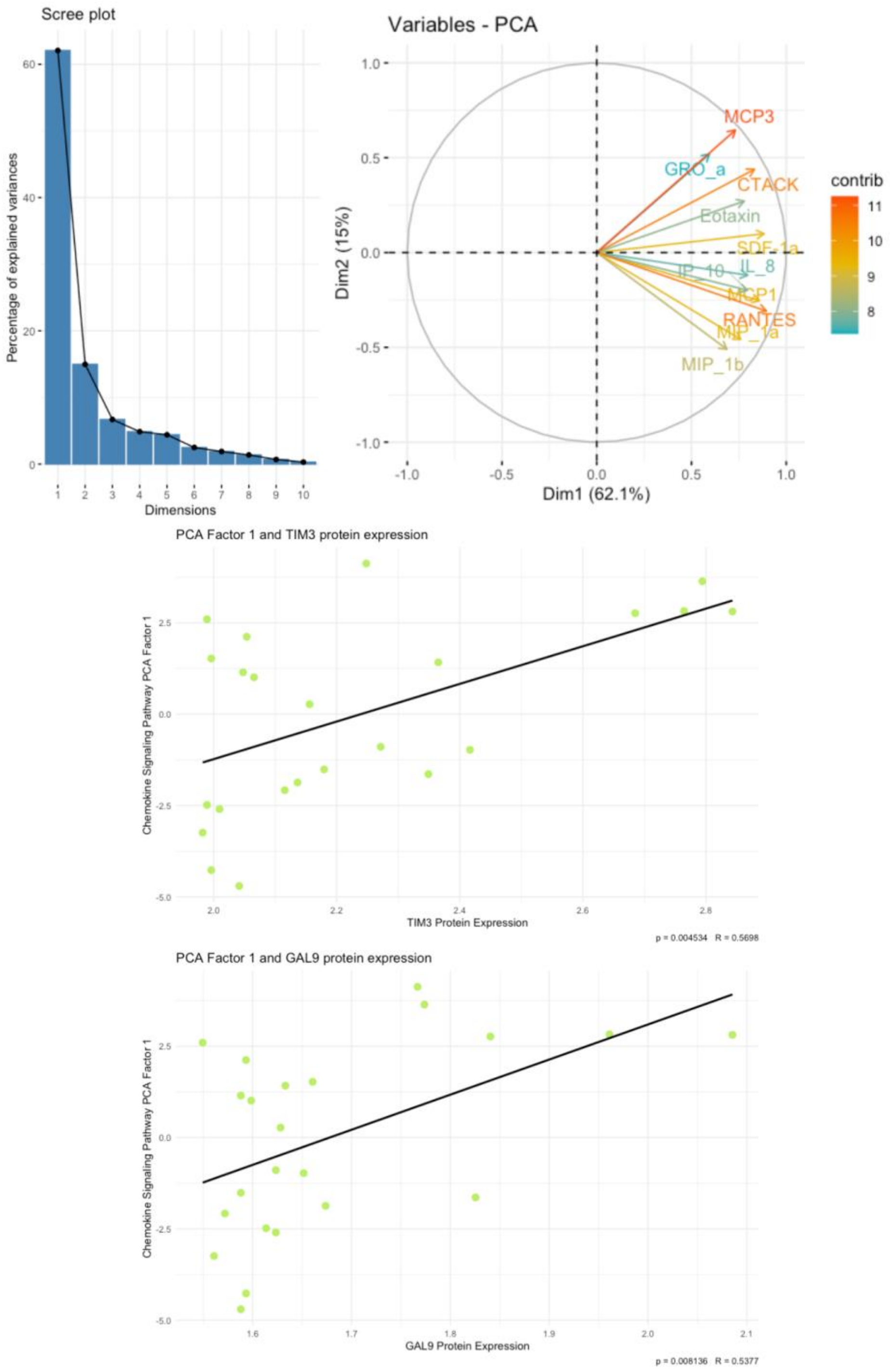

Figure S2. Scree plot, biplot, and correlation plot for chemokine signaling pathway set of cytokines and TIM-3, and Gal-9 expression. The green dots on the plot represent data points from PCA used in the analysis of the correlation between PCA Factor 1 and the TIM-3 and Gal-9 protein expressions.

	eigenvalue	Variance (%)	Cumulative variance (%)
PCA Factor 1	6.994754256	43.71721410	43.71721
PCA Factor 2	2.390928191	14.94330119	58.66052
PCA Factor 3	1.799793275	11.24870797	69.90922

Table S7. Eigenvalue and the percentage of explained variance for 3 factors (principal components) from the PCA for KEGG IL-17 signaling pathway.

Variable	Factor 1	Factor 2	Factor 3
Positive regulation of lymphocyte chemotaxis			
MCP1	0.6981426	0.36667237	0.47960128
IL_8	0.7754460	0.06992977	0.33240235
IL_1b	0.4653809	-0.45436377	0.35541625
IL_4	0.8979991	-0.10481841	-0.05887591
IL_5	0.4503014	-0.69547799	-0.38385927
IL_6	0.5024565	0.43113264	-0.31836801
IL_13	0.1972706	0.66210070	-0.44558392
IL_17	0.6369006	0.69756299	-0.17812979
GM_CSF	0.6828084	0.01533992	-0.47693633
IFN_g	0.3215045	-0.50092628	0.33966848
G_CSF	0.5756592	0.73097713	0.10434901
TNF-a	0.8994702	-0.08067274	-0.16853841
MCP3	0.8895354	-0.19880433	-0.29306966
Eotaxin	0.8023776	-0.10888111	0.20023469
GRO_a	0.7001830	-0.07656198	-0.19456378
IP_10	0.6248622	0.24878612	0.56022254

Table S8. Loadings of 3 factors (PCA Factors) after varimax rotation. Coordinates for the variables for KEGG IL-17 signaling pathway.

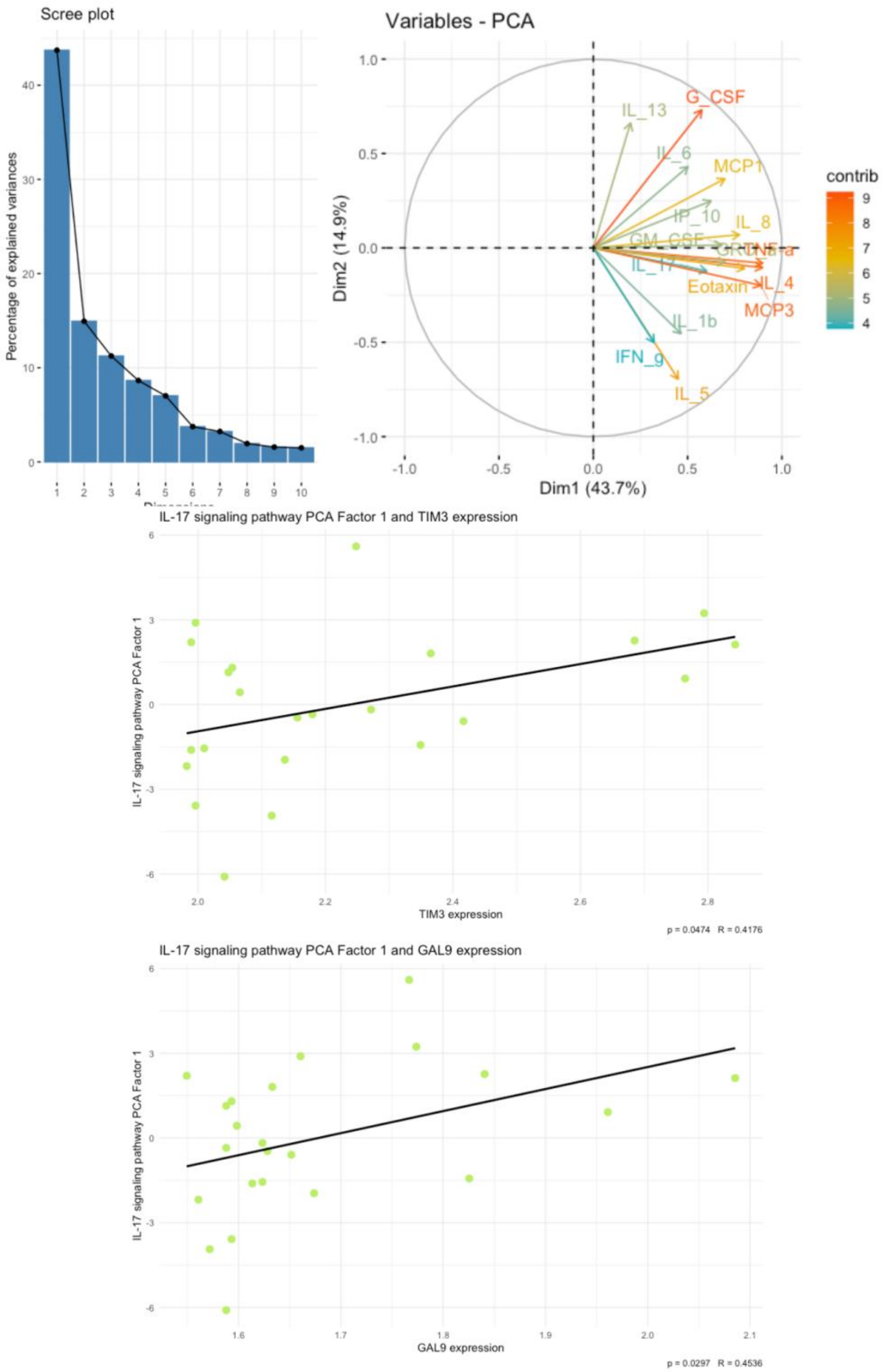

Figure S3. Scree plot, biplot, and correlation plot for KEGG IL-17 signaling pathway *set of cytokines* and TIM-3, and Gal-9 expression. The green dots on the plot represent data points from PCA used in the analysis of the correlation between PCA Factor 1 and the TIM-3 and Gal-9 protein expressions.

	eigenvalue	Variance (%)	Cumulative variance (%)
PCA Factor 1	5.16453498	57.3837220	57.38372

PCA Factor 2	0.94406805	10.4896450	67.87337
PCA Factor 3	0.89253253	9.9170281	77.79040

Table S9. Eigenvalue and the percentage of explained variance for 3 factors (principal components) from the PCA for NOD-like receptor signaling pathway set of cytokines.

Variable	Factor 1	Factor 2	Factor 3
Positive regulation of lymphocyte chemotaxis			
IL_8	0.8431128	-0.20619214	0.3321359
TNF-a	0.8302601	-0.09082199	-0.3396636
MCP1	0.7915168	0.34377348	0.1497477
IFN_a2	0.8364194	0.03494157	-0.4138619
GRO_a	0.6479344	-0.01139049	-0.5230681
IL_6	0.5744879	0.01629259	0.3215926
IL_1b	0.4910675	-0.82141595	0.1217876
RANTES	0.8359188	0.28827703	0.2544498
IL_18	0.8651617	0.12522019	0.1286152

Table S10. Loadings of 3 factors (PCA Factors) after varimax rotation. Coordinates for the variables for NOD-like receptor signaling pathway set of cytokines.

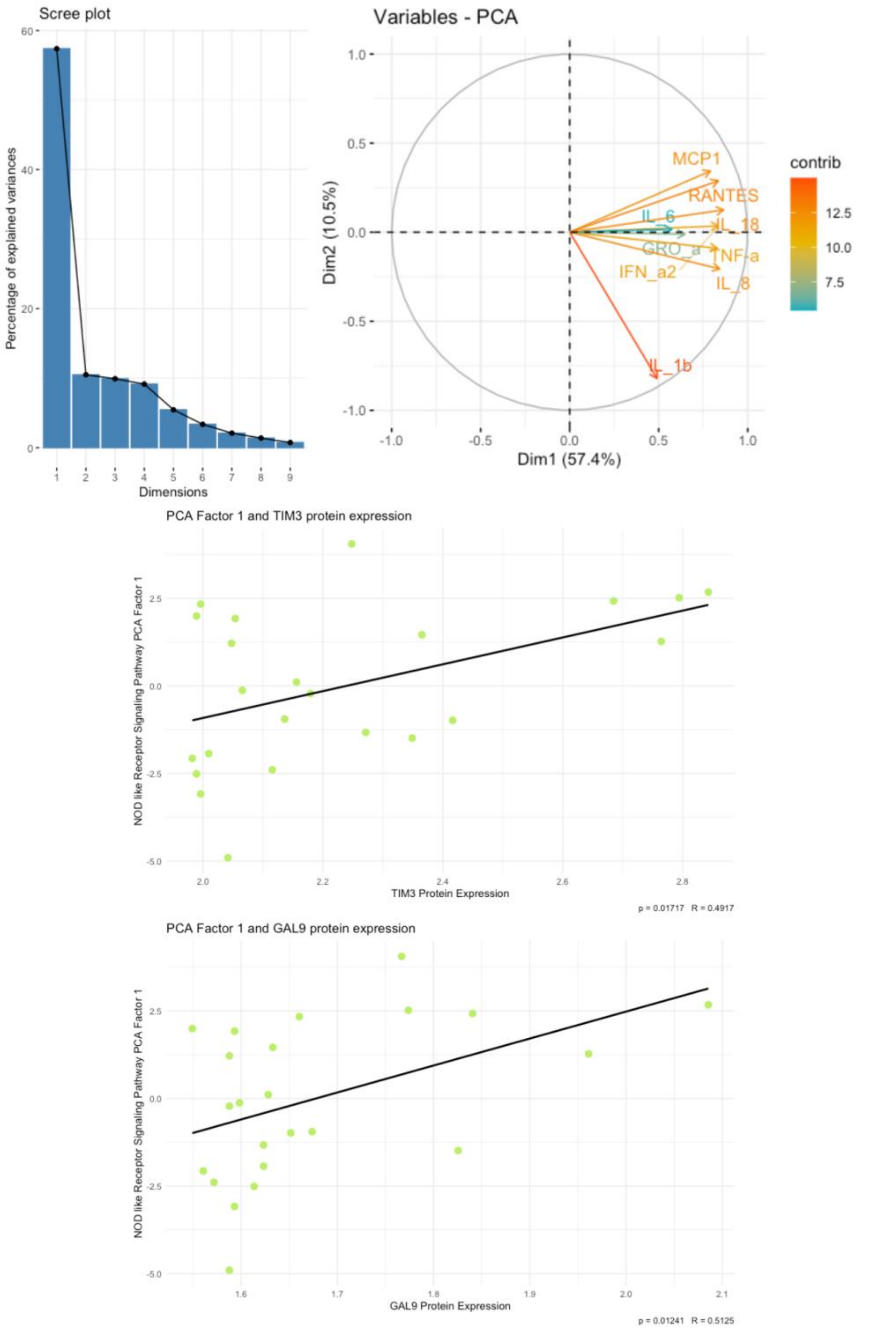

Figure S4. Scree plot, biplot, and correlation plot for NOD-like receptor signaling pathway set of cytokines and TIM-3, and Gal-9 expression. The green dots on the plot represent data points from PCA used in the analysis of the correlation between PCA Factor 1 and the TIM-3 and Gal-9 protein expressions.

	eigenvalue	Variance (%)	Cumulative variance (%)
PCA Factor 1	5.54340953	61.5934392	61.59344

PCA Factor 2	1.43233790	15.9148656	77.50830
PCA Factor 3	0.68507169	7.6119076	85.12021

Table S11. Eigenvalue and the percentage of explained variance for 3 factors (principal components) from the PCA for Macrophage chemotaxis set of cytokines.

Variable	Factor 1	Factor 2	Factor 3
Positive regulation of lymphocyte chemotaxis			
MCP1	0.8836440	-0.12309642	0.28290478
IL_8	0.8474851	-0.07224071	-0.29658301
Eotaxin	0.7447201	0.34238929	-0.40400847
MIG	0.8316331	-0.38028266	0.07361772
IP_10	0.8273643	-0.06412701	0.31812974
GRO_a	0.5806476	0.63019397	0.33072499
MIP_1b	0.7254812	-0.47141995	-0.29993071
MCP3	0.6369006	0.69756299	-0.17812979
RANTES	0.9189567	-0.20008379	0.12711599

Table S12. Loadings of 3 factors (PCA Factors) after varimax rotation. Coordinates for the variables for Macrophage chemotaxis set of cytokines.

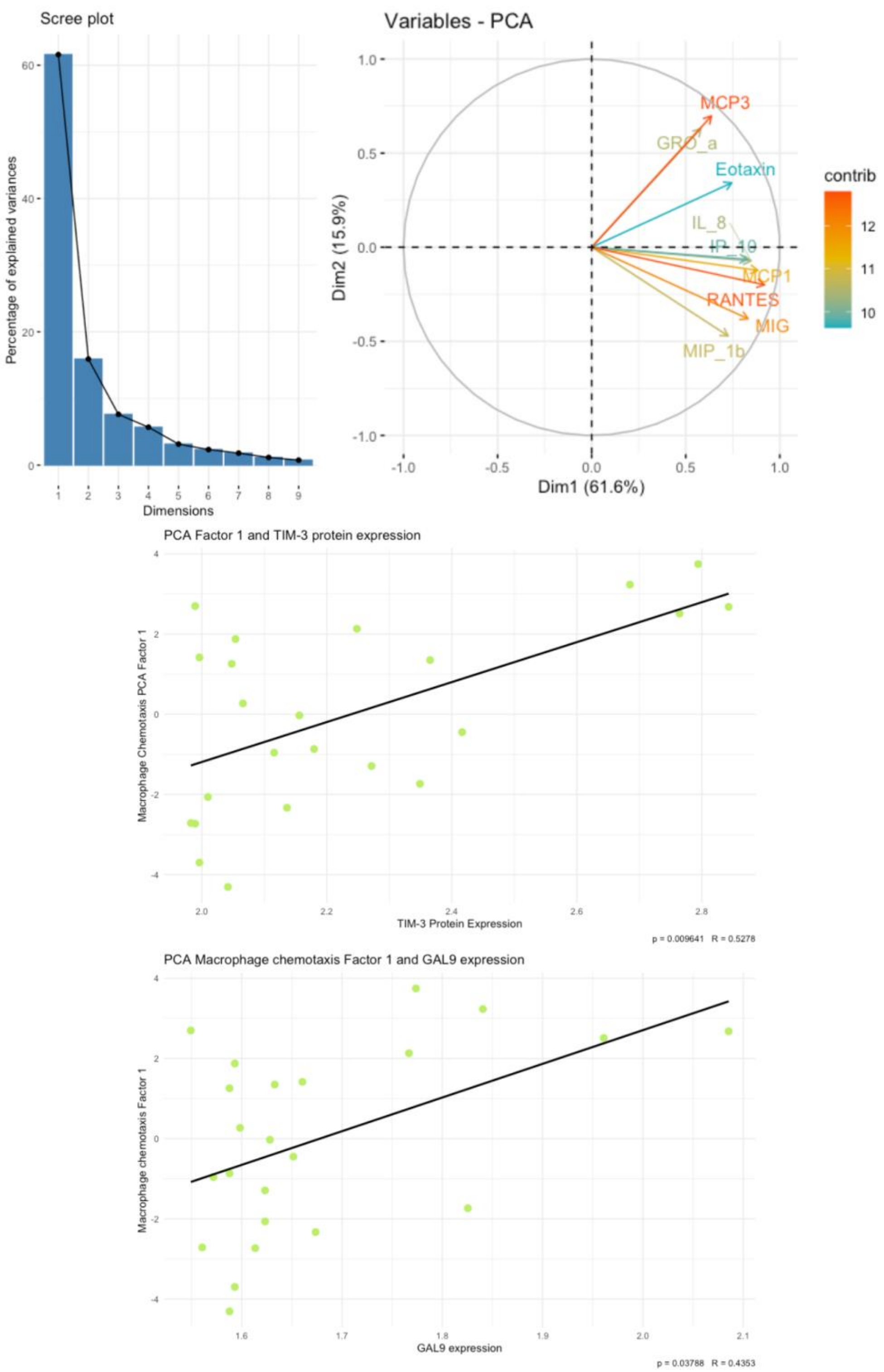

Figure S5. Scree plot, biplot, and correlation plot for Macrophage chemotaxis *set of cytokines* and TIM-3, and Gal-9 expression. The green dots on the plot represent data points from PCA used in the analysis of the correlation between PCA Factor 1 and the TIM-3 and Gal-9 protein expressions.

	eigenvalue	Variance (%)	Cumulative variance (%)
PCA Factor 1	4.62488887	66.069841	66.06984

PCA Factor 2	1.27826321	18.260903	84.33074
PCA Factor 3	0.51702058	7.386008	91.71675

Table S13. Eigenvalue and the percentage of explained variance for 3 factors (principal components) from the PCA for Positive regulation of lymphocyte migration set of cytokines.

Variable	Factor 1	Factor 2	Factor 3
Positive regulation of lymphocyte chemotaxis			
IP_10	0.7859457	-0.1868771	0.56031266
SDF-1a	0.9274965	0.1866401	-0.07481630
MIP_1a	0.7951341	-0.4276187	-0.14856731
CTACK	0.8232000	0.5252618	-0.09799079
MIP_1b	0.7219227	-0.4574863	-0.37966857
MCP3	0.6996288	0.6764789	-0.03877205
RANTES	0.9090540	-0.2878029	0.14193974

Table S14. Loadings of 3 factors (PCA Factors) after varimax rotation. Coordinates for the variables for Positive regulation of lymphocyte migration set of cytokines.

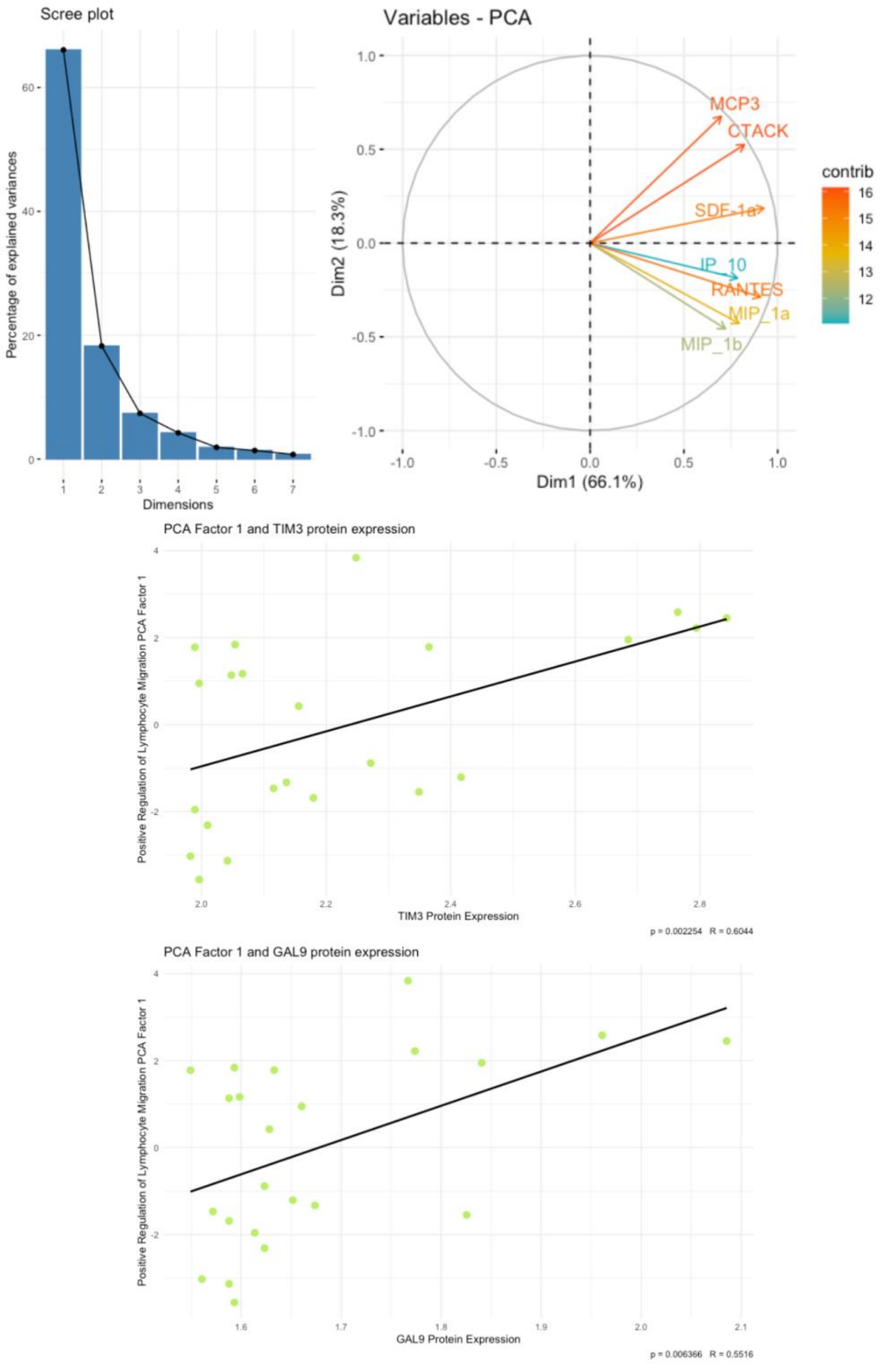

Figure S6. Scree plot, biplot, and correlation plot for Positive regulation of lymphocyte migration set of cytokines and TIM-3, and Gal-9 expression. The green dots on the plot represent data points from PCA used in the analysis of the correlation between PCA Factor 1 and the TIM-3 and Gal-9 protein expressions.

	eigenvalue	Variance (%)	Cumulative variance (%)
PCA Factor 1	6.19026114	47.6173934	47.61739

PCA Factor 2	2.15087794	16.5452149	64.16261
PCA Factor 3	1.29829783	9.9869063	74.14951

Table S15. Eigenvalue and the percentage of explained variance for 3 factors (principal components) from the PCA for protumor cytokines subgroup with TIM-3 protein expression.

Variable	Factor 1	Factor 2	Factor 3
Positive regulation of lymphocyte chemotaxis			
IL_1a	0.55725313	0.61959643	-0.17888010
IL_1b	0.46795470	0.40602433	-0.69998553
IL_6	0.60310784	-0.16032835	0.23397994
IL_8	0.83152146	-0.02369018	-0.27701885
IL_17	0.61296169	0.27842081	-0.20841291
IL_18	0.89149891	-0.14471440	0.07053479
TNF-a	0.86760259	0.30265952	0.16760992
RANTES	0.80316092	-0.44575991	-0.19330756
MCP1	0.71598463	-0.45165297	-0.17352801
MCP3	0.78253602	0.29981170	0.32572849
MIF	0.05820564	-0.72397993	-0.13497325
G_CSF	0.69466507	-0.55953713	0.10618946
GM_CSF	0.65294974	0.23661098	0.60436491

Table S16. Loadings of 3 factors (PCA Factors) after varimax rotation. Coordinates for the variables for protumor cytokines subgroup.

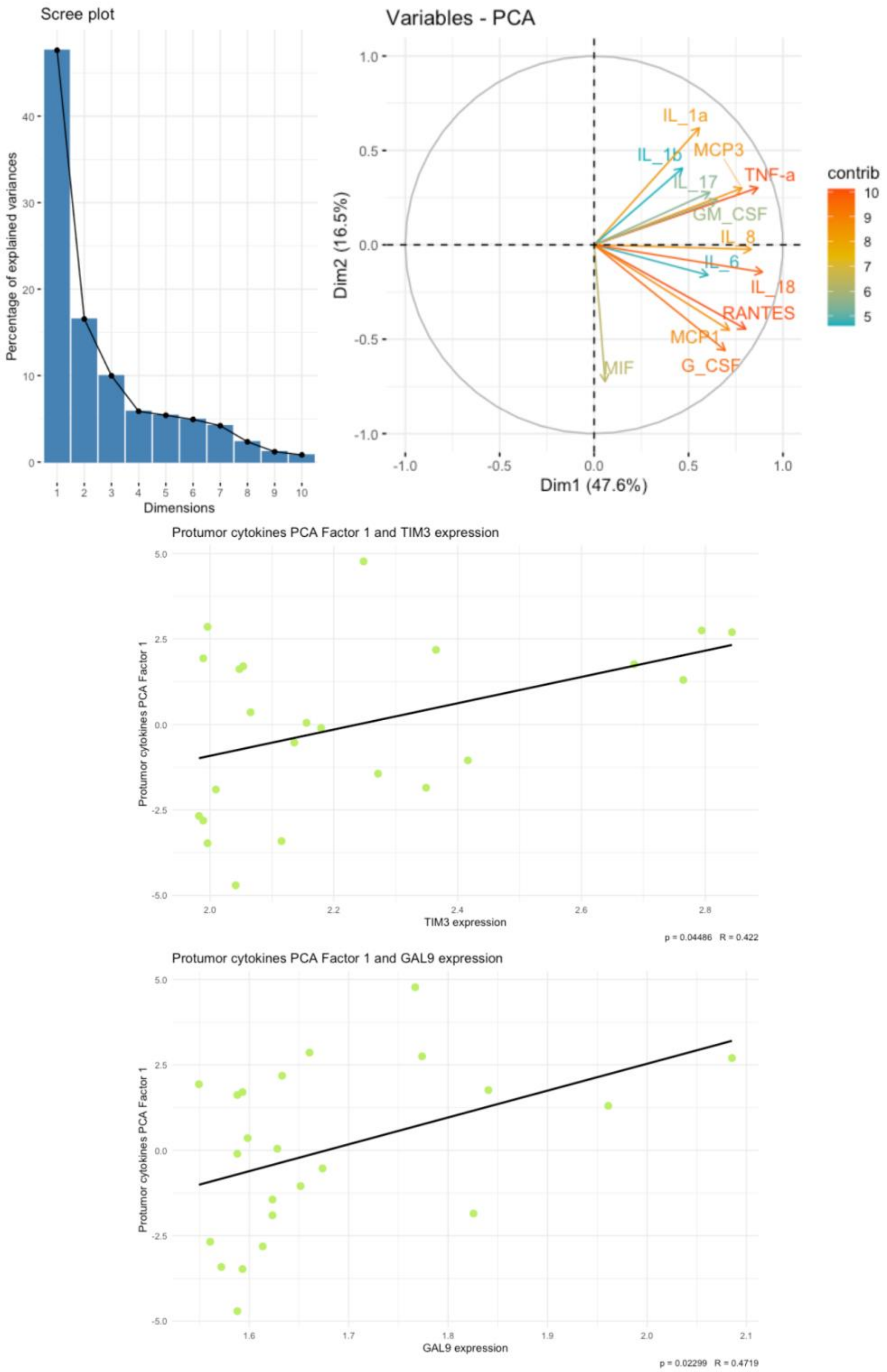

Figure S7. Scree plot, biplot, and correlation plot for protumor cytokines subgroup and TIM-3, and Gal-9 expression. The green dots on the plot represent data points from PCA used in the analysis of the correlation between PCA Factor 1 and the TIM-3 and Gal-9 protein expressions.

Pathway	GAL9 ϱ	GAL9 p-value	GAL9 q-value	GAL9 FDR < 0.05	TIM-3 ϱ	TIM-3 p-value	TIM-3 q-value	TIM-3 FDR < 0.05
Chemokine signaling	0.538	0.0081	0.0244	Yes	0.57	0.0045	0.0136	Yes
Interleukin-10 signaling	0.504	0.0143	0.0429	Yes	0.481	0.0202	0.0606	No
Interleukin-17 signaling pathway	0.454	0.0297	0.0891	No	0.418	0.0474	0.1422	No
NOD-Like receptor signaling pathway	0.512	0.0124	0.0372	Yes	0.492	0.0172	0.0515	No
Macrophage chemotaxis	0.435	0.0379	0.1136	No	0.528	0.0096	0.0289	Yes
Positive regulation of lymphocyte migration	0.552	0.0064	0.0191	Yes	0.604	0.0023	0.0068	Yes
Protumor cytokines	0.472	0.023	0.069	No	0.422	0.0449	0.1346	No

Table S17. Spearman Correlations Between First Principal Component (PC1) of Immune Pathway-Specific PCA cytokine subsets and Tumor Expression of GAL9 and TIM-3 With FDR Correction.

	Tumor grading	Primary tumor localization	MSI status
	p-value	p-value	p-value
TIM-3	0.7002	0.579	0.9488
GAL9	0.7751	0.586	0.3101

Table S18. TIM-3 and Gal-9 proteins concentration and tumor grading, primary tumor localization, and MSI status. scale parameters, tumor stage, and TILs. p-value was derived from the Mann–Whitney U test for TIM-3 and Gal-9 proteins concentration.

	T parameter		N parameter		M parameter	Tumor Stage		Tumor Infiltrated Lymphocytes (TILs)	
	p-value	tau	p-value	tau	p-value	p-value	tau	p-value	tau
TIM-3	0.6353	-0.0327	0.1071	0.1107327	0.2654	0.1342	0.1001714	0.8995	0.01096112

GAL9	0.9768	-0.0020	0.125	0.105828	0.6564	0.6744	0.02821043	0.818	0.02012643

Table S19. TIM-3 and Gal-9 protein concentration and TNM scale parameters, tumor stage, and TILs. Kendall's Tau rank correlation coefficient p-value and Tau for T and N parameters, tumor stage, and TILs for TIM-3 ang Gal-9 expression. p-value for the M parameter was derived from the Mann–Whitney U test.
